# Quest for optimal treatment of cluster-C personality disorders: evaluating schema therapy formats in a multicentre randomised controlled trial

**DOI:** 10.1136/bmjment-2026-302929

**Published:** 2026-10-26

**Authors:** Anne-Sophie S M Venhuizen, Iuno Z Groot, Raoul P P P Grasman, Silvia M A A Evers, Joël R Van Aalderen, Mascha Nuijten, Elvan Tutkun, Susanne Dalmeijer, Kasper Nikkels, Myrte Maarschalkerweerd, Charlotte M E Van Ginkel, Agatha Ph Hollander, Fatima Jegić, Lotte Timmerman, Nathan Bachrach, Arnoud Arntz

**Affiliations:** 1 Department of Clinical Psychology, University of Amsterdam, Amsterdam, The Netherlands; 2 Department of Psychological Methods, University of Amsterdam, Amsterdam, The Netherlands; 3 Department of Health Services Research, Care and Public Health Research Institute (CAPHRI), Maastricht University, Maastricht, The Netherlands; 4 Trimbos Institute, Utrecht, The Netherlands; 5 PsyQ Utrecht, Utrecht, The Netherlands; 6 PsyQ Zaandam, Zaandam, The Netherlands; 7 PsyQ Amsterdam, Amsterdam, The Netherlands; 8 PsyQ Rotterdam, Rotterdam, The Netherlands; 9 Academic Centre for Trauma and Personality, Amsterdam, The Netherlands; 10 IPGGZ Groningen, Groningen, The Netherlands; 11 IPGGZ Veendam, Veendam, The Netherlands; 12 Emergis, Goes, The Netherlands; 13 Department of Medical and Clinical Psychology, Tilburg University, Tilburg, The Netherlands; 14 Department of Personality Disorders, GGZ Oost Brabant, Helmond, The Netherlands

**Keywords:** Personality Disorders, Schema Therapy

## Abstract

**Background:**

Schema therapy (ST) is proven to be effective for cluster-C personality disorders (PDs). Recently, a group format of ST (GST) has gained popularity but has not been compared with other treatments.

**Objective:**

To compare the effectiveness of GST to individual ST (IST) and specialised treatment as usual (sTAU) for cluster-C PDs.

**Method:**

We conducted a multicentre parallel-group, randomised controlled trial (QUalitative and Effectiveness Study to select optimal Treatment for Cluster-C PD) comparing 1 year of GST and IST with open-ended sTAU, with a follow-up assessment 2 years after start of treatment. Outpatients (>18 years) with a primary cluster-C PD were recruited and assigned to GST (30 weekly sessions plus 4 monthly booster sessions), IST (50 sessions with decreasing frequency) or sTAU (routine care in PD-specialised departments). The primary outcome was cluster-C PD severity change.

**Results:**

369 participants (mean (SD) age, 37.24 (11.21) years; 242 (65.6) female) received GST (46.6%), IST (26.0%) or sTAU (27.4%). At 1 year, GST was superior to sTAU (d=0.33, 95% CI 0.05 to 0.61, p=0.02), and IST was superior to sTAU (d=0.41, 95% CI 0.10 to 0.72, p=0.010). GST and IST did not differ significantly (d=0.08, 95% CI −0.19 to 0.35, p=0.57). No significant differences were found at 2 years (GST-sTAU: d=0.12, 95% CI −0.18 to 0.42, p=0.44; GST-IST: d=0.11, 95% CI −0.18 to 0.39, p=0.46; IST-sTAU: d=0.23, 95% CI −0.11 to 0.56, p=0.18). Treatment retention in IST (92.7%) was superior to sTAU (78.2%) but did not differ significantly with GST (84.9%) at 1 year. At 2 years, treatment retention in IST and GST remained the same and was both superior to sTAU (72.3%).

**Conclusion:**

Both GST and IST outperformed sTAU after 1 year, although differences were no longer evident at follow-up. GST and IST showed higher retention rates than sTAU.

**Clinical implications:**

These findings support GST and IST as good treatment options for cluster-C PDs.

**Trial registration number:**

NL-OMON19942.

**WHAT IS ALREADY KNOWN ON THIS TOPIC**

- Cluster-C personality disorders (PDs) are highly prevalent and are accompanied by a significant burden, but research into evidence-based treatment options is limited.
- Individual schema therapy (IST) is proven to be an effective treatment for cluster-C PDs. A new group format of ST (GST) is popular in clinical practice but has not yet been compared with other psychotherapies for this patient population.

**WHAT THIS STUDY ADDS**

- This randomised controlled trial showed that GST and IST were both significantly more effective than specialised treatment as usual at 1 year of treatment, but there were no significant differences 2 years after the start of treatment.

**HOW THIS STUDY MIGHT AFFECT RESEARCH, PRACTICE OR POLICY**

- The findings show no long-term superiority of either GST, IST or sTAU for patients with cluster-C PD and indicate that both ST formats can be more effective in the short term than sTAU.

## Background

 Individuals with cluster-C personality disorders (PDs), including avoidant, dependent and obsessive-compulsive PD, comprise a large group of patients who have thus far been under-represented in research. This is problematic, as the numbers of patients are vast (up to 30% in clinical populations^1^), and the suffering of this population is substantial. Recent studies show large impairments in quality of life and functioning and high societal costs^2 3^ comparable to or only slightly lower than those of borderline PD.^4^ To provide this population with the necessary care, it is imperative to conduct more studies into the effectiveness of interventions.

Schema therapy (ST), developed by Young^5^ to treat chronic and persistent psychological disorders more effectively, appears promising for cluster-C PDs. Central to ST are early maladaptive schemas (EMS) that individuals develop about themselves, others and the world and are thought to be rooted in unmet childhood needs. When triggered, individuals can cope with the EMS and accompanying negative emotions either adaptively or maladaptively (avoiding, surrender and overcompensating), resulting in momentary emotional-cognitive-behavioural states known as schema modes. Specific PD-schema mode models are developed for cluster-C PDs,^6^ as a framework to understand their complex and pervasive problems. These personalised schema mode models guide treatment, focusing on recognising emotional needs, reducing maladaptive and enhancing positive schema modes. ST has proven to be effective for several complex disorders, including borderline PD.^7–9^ A randomised controlled trial (RCT) showed that individual ST (IST) was more (cost-)effective than clarification-oriented psychotherapy (COP) and specialised treatment as usual (sTAU) for several PDs, including cluster-C PDs.^10 11^

Recently, group formats of ST (GST) gained popularity, primarily due to their potential to treat more patients with fewer therapists. Moreover, the group format may provide additional therapeutic benefits, such as peer support, opportunities for practising social interactions in vivo and vicarious learning through observing others.^12 13^ Preliminary evidence supporting GST’s effectiveness was provided by a pilot study^14^ and corroborated by a multicentre trial.^15^ Additionally, an RCT compared GST with group cognitive behavioural therapy in individuals with social anxiety disorder and comorbid avoidant PD.^16^ The treatments didn’t differ in effectiveness; however, GST had a lower dropout rate. Although the results for GST are promising, a possible disadvantage of group formats is that clients receive less individual time, which may be needed for treating the persistent nature of cluster-C PDs. Additionally, although a group format can be especially effective in eliciting and addressing maladaptive patterns of avoidance and anxiety, this same format may still pose a significant challenge for individuals who are introverted or highly socially anxious. This could lead some participants to withdraw, become less vocal or focus too much on others rather than their own recovery. Evidence for the surplus value of group over individual therapy in cluster-C PDs remains inconclusive. However, an RCT in borderline PD showed greater symptom reduction in combined IST-GST than in predominantly GST and sTAU, suggesting that adding IST to GST has benefits.^17^

### Objective

Thus far, no comparison has been made between the group and individual format of ST for cluster-C PD. Therefore, this study compares the effectiveness of GST to sTAU and IST and explores differences between IST and sTAU. The primary outcome was the decrease in the severity of cluster-C PD symptoms. Secondary outcomes included treatment retention, general functioning, psychopathology, quality of life, happiness, PD-related beliefs, self-esteem, self-ideal discrepancy, introversion, schemas, schema modes and sleep problems.

## Methods

### Study design

The QUalititative and Effectiveness Study to select optimal Treatment for Cluster-C PD (QUEST-CLC) is a multicentre, parallel-group, superiority RCT comparing the effectiveness of GST versus sTAU and IST; see the design paper for further details.^18^ This trial is registered at the Overview of Medical Research in the Netherlands (OMON; ID: NL-OMON19942) at https://trialsearch.who.int/Trial2.aspx?TrialID=NL-OMON19942

### Participants

Individuals with a cluster-C PD were recruited from the regular influx of 10 Dutch mental health centres between the 19th of March 2021 and June 2023 (final follow-up assessment took place in April 2025): GGZ-Oost Brabant sites Oss, Boxmeer and Helmond, IPGGZ sites Groningen and Veendam, Emergis, PsyQ sites Utrecht, Rotterdam, Zaandam and Amsterdam. The PsyQ Amsterdam site was closed in February 2023; clients and therapists were transferred to the Academic Centre for Trauma and Personality, where recruitment and treatment continued. Inclusion criteria consisted of (1) primary cluster-C PD diagnosis as determined by the Structured Clinical Interview for Diagnostic and Statistical Manual of Mental Disorders fifth edition Personality Disorders (SCID-5-PD)^19^, (2) age ≥18 years and (3) motivation and availability to participate in 1-year treatment. Patients were excluded if they (1) had acute suicidality or (2) had received ST in the previous year. The power analyses determined a sample size of 378 participants (power 0.90, p=0.025, d=0.44, see the study protocol^18^ for how d=0.44 was estimated). A slightly smaller sample was recruited, n=375, still yielding 0.89 power.

### Randomisation

Computerised randomisation, stratified by primary cluster-C diagnosis and gender to ensure equal distribution over the three treatment conditions, was conducted by an independent researcher at the University of Amsterdam. Per site, participants were randomly allocated to GST, IST and sTAU in a 3:2:2 ratio. Randomisation took place every 10 weeks to ensure enough enrolment in GST. Participants were informed of the randomisation outcome in the first treatment session of the assigned treatment arm.

### Treatments

GST followed the Tjoa and Muste protocol,^20^ which is based on the format established by Farrell and Shaw.^21^ GST comprised 30 weekly group sessions of 90 min, supplemented by 4 monthly booster sessions (60 min) in smaller groups with a single therapist. Additionally, patients could use up to 300 min of optional individual sessions whenever they felt it was needed. Every 10 sessions, new participants entered and those completing 30 sessions left the group. Each group was led by two therapists and generally consisted of 5–9 patients. IST followed the 50-session protocol of Arntz.^6^ Sessions lasted 45–50 min and were delivered with decreasing frequency: 15 weeks of twice-weekly sessions, followed by 15 weekly sessions and five booster sessions over 4 months. ST patients were asked to follow only the study treatment and postpone any follow-up treatment until after the last assessment, except for crisis interventions if necessary. Adherence to this advice was only assessed at follow-up. All ST therapists were experienced and fulfilled the study’s training requirements (see study protocol^18^). sTAU was designed to mimic standard specialised treatment that patients with cluster-C PDs receive in Dutch treatment centres with specialised PD departments. Participating PD departments offered their regular treatment for patients with cluster-C PD with the exclusion of treatments incorporating ST techniques. sTAU included a variety of specialised treatments, such as guideline-informed treatment for personality disorders (GITPD) and short-term schema-based cognitive behavioural (SCB) group therapy. A detailed description of the treatments is in the study protocol^18^ and an overview of sTAU treatments in online supplemental sTable 1. Therapy sessions were video-recorded to assess treatment integrity. IST and GST showed significantly higher ST adherence (d=2.5, 1.3) and ST competence (d=1.7, 0.8) than individual and group sTAU. Facilitative conditions and explicit directiveness were also higher in IST/GST than in sTAU (see online supplement 2 and sTable 2).

### Assessments

Five assessments were administered by blinded assessors: baseline (0 weeks), mid-treatment (17 weeks), post-treatment (34 weeks), post-booster (51 weeks) and follow-up (104 weeks). When participants had to wait longer than 2.5 months to start treatment, the baseline was reassessed. After completion of post-booster and follow-up assessments, participants received 20 euros.

### Outcome measures

The primary outcome was severity of the primarily diagnosed PD during the last month, measured using diagnosis-specific interviews: Avoidant Personality Disorder Severity Index,^22^ Obsessive-Compulsive Personality Disorder Severity Index^23^ and Dependent Personality Disorder Severity Index.^24^ Scores of the three indices were standardised using the baseline SD. See online supplement 3 for descriptions and psychometric properties of primary and secondary outcomes.

### Data analysis

In line with the intention-to-treat principle, analyses were performed on data from all randomised participants who started treatment. Differences between treatments in change over time were analysed with linear mixed regression models (LMM) for normally distributed data and generalised LMM (GLMM) gamma regression with a log link for skewed data. These models allow for dealing with missing observations and nested data (assessments within patients, inclusion cohorts and sites). To model the repeated part, an AR1 covariance structure was used (online supplement 4). Fixed effects of the models consisted of time, treatment and their interactions, with random effects for site, inclusion cohort and participant if it improved model fit and if the fitting algorithm could be made to converge without problems (online supplement 4). The time model was three-piecewise due to inflection points observed in the raw data at post-treatment and post-booster and superior fits as indicated by the Akaike information criterion and Bayesian information criterion. Additionally, patients in the ST arms had completed treatment at the post-treatment assessment, but for sTAU this was often not the case, making an additional comparison at post-booster equitable.

Estimated means with 95% CIs were calculated for the three treatments over time. Cohen’s d was calculated for fixed effects (online supplement 5). For the primary outcome, a Reliable Change Index was calculated (online supplement 6), and several sensitivity analyses were conducted. These included the addition of the following predictors to the model: the primary cluster-C diagnosis (online supplement 7, online supplemental sTable 5 and online supplemental sFigures 1 and 2), demographic variables (online supplement 8), missingness at follow-up (online supplement 9) and (number of) treatment sessions after the post-booster assessment (online supplement 10 and sTable 6). For all outcome variables, analyses were rerun without outliers and influential cases and reported when findings differed. Treatment retention was analysed as time till dropout in the first and second year by Kaplan-Meier survival analysis with log-rank tests. Analyses were blinded for treatment type (online supplement 1) and performed in R (V.4.3.2) and in IBM SPSS (V.29). A two-tailed p-value of <0.025 for the primary outcome and p<0.05 for all other analyses was set to determine statistical significance.

## Findings

The Consolidated Standards of Reporting Trials (CONSORT) diagram of participant flow and reasons for exclusion or drop-out is shown in figure 1. Table 1 presents baseline statistics of the 369 patients that participated in the study. Results for the primary and secondary outcome variables can be found in table 2 and online supplemental sTables 3 and 4.

**Figure 1:**
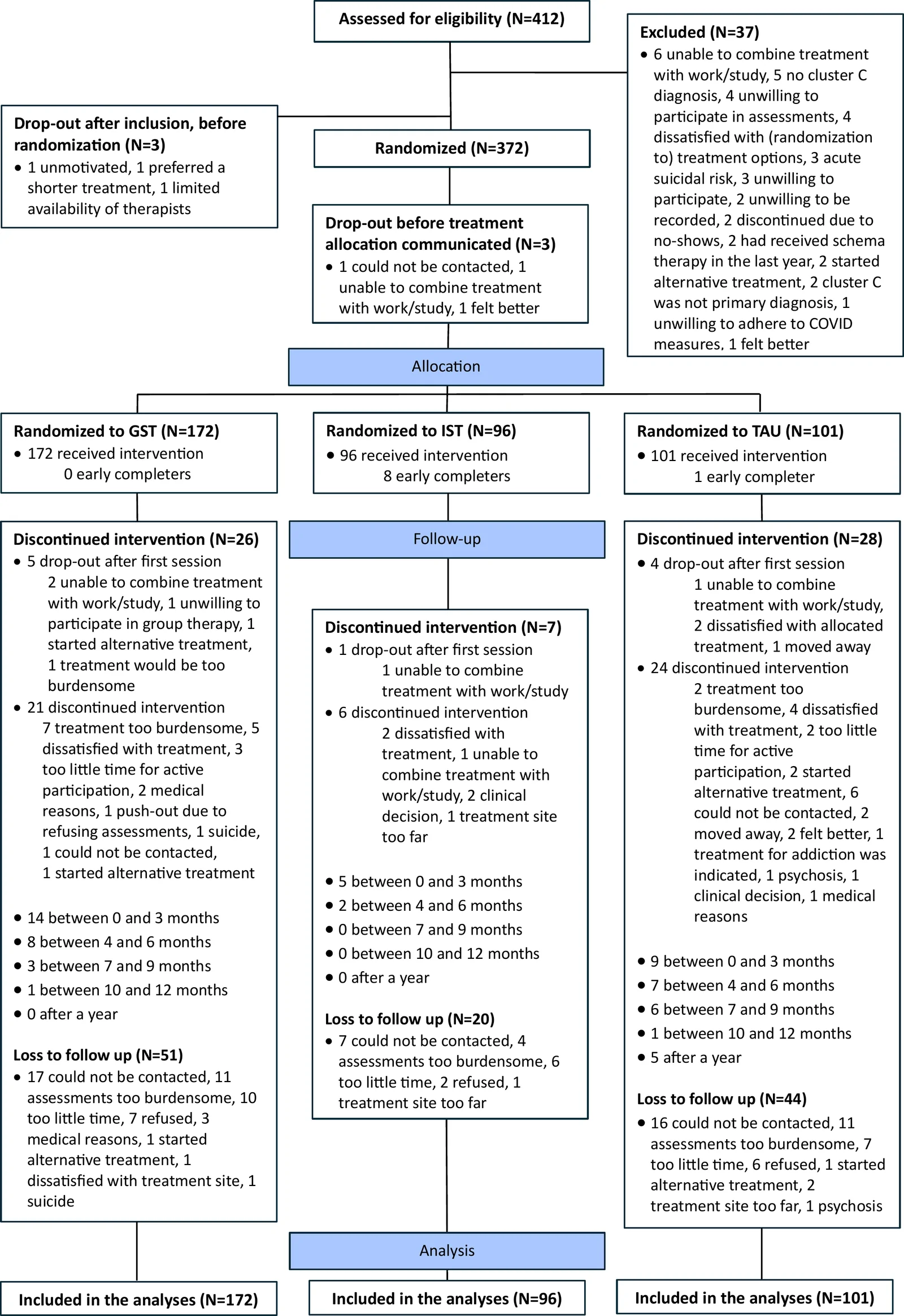
Consolidated Standards of Reporting Trials (CONSORT) flow diagram. GST, group schema therapy; IST, individual schema therapy; sTAU, specialised treatment as usual.

**Table 1:** Demographic and clinical characteristics of participants at baseline

Characteristic*	GST (n=172)	IST (n=96)	sTAU (n=101)	Total (n=369)
Age, mean (SD), years	36.55 (11.21)	38.72 (10.94)	37.01 (11.44)	37.24 (11.21)
Gender				
Female	110 (64.0)	62 (64.6)	70 (69.3)	242 (65.6)
Male	62 (36.0)	33 (34.4)	30 (29.7)	125 (33.9)
Other	0 (0)	1 (1.0)	1 (1.0)	2 (0.5)
Living situation				
Alone	60 (34.9)	34 (35.4)	46 (45.5)	140 (37.9)
With partner	104 (60.5)	61 (63.5)	49 (48.5)	214 (58.0)
With parents/other(s)	8 (4.7)	1 (1.0)	6 (5.9)	15 (4.1)
Ethnic background				
Dutch	144 (83.7)	84 (87.5)	71 (70.3)	299 (81.0)
Partially Dutch	19 (11)	5 (5.2)	12 (11.9)	36 (9.8)
Non-Dutch	9 (5.2)	7 (7.3)	18 (17.8)	34 (9.2)
Education†				
Early childhood	1 (0.6)	0 (0)	0 (0)	1 (0.3)
Primary	8 (4.7)	2 (2.1)	3 (3.0)	13 (3.5)
Lower secondary	13 (7.6)	8 (8.3)	9 (8.9)	30 (8.1)
Upper secondary	72 (41.9)	43 (44.8)	51 (50.5)	166 (45.0)
Bachelor	53 (30.8)	26 (27.1)	26 (25.7)	105 (28.5)
Master	25 (14.5)	17 (17.7)	12 (11.9)	54 (14.6)
Employment status				
Working	76 (44.2)	53 (55.2)	40 (39.6)	169 (45.8)
Sick leave	26 (15.1)	15 (15.6)	10 (9.9)	51 (13.8)
Disability	30 (17.4)	11 (11.5)	26 (25.7)	67 (18.2)
Welfare	10 (5.8)	8 (8.3)	8 (7.9)	26 (7.0)
Unemployed	13 (7.6)	3 (3.1)	6 (5.9)	22 (6.0)
Student	17 (9.9)	6 (6.3)	11 (10.9)	34 (9.2)
Primary personality disorder				
Avoidant	127 (73.8)	74 (77.1)	82 (81.2)	283 (76.7)
Obsessive-compulsive	40 (23.3)	19 (19.8)	17 (16.8)	76 (20.6)
Dependent	5 (2.9)	3 (3.1)	2 (2.0)	10 (2.7)
Comorbid personality disorder				
Dependent	4 (2.3)	1 (1.0)	1 (1.0)	6 (1.6)
Obsessive-compulsive	8 (4.7)	9 (9.4)	7 (6.9)	24 (6.5)
Avoidant	8 (4.7)	3 (3.1)	3 (3.0)	14 (3.8)
Borderline	2 (1.2)	4 (4.2)	4 (4.0)	10 (2.7)
Narcissistic	0 (0)	0 (0)	0 (0)	0 (0)
Histrionic	1 (0.6)	0 (0)	0 (0)	1 (0.3)
Paranoid	2 (1.2)	0 (0)	0 (0)	2 (0.5)
None	148 (86)	79 (82.3)	87 (86.1)	314 (85.1)
Comorbid syndrome disorder				
None	33 (19.2)	21 (21.9)	16 (15.8)	70 (19.0)
Anxiety	79 (45.9)	42 (43.8)	46 (45.5)	167 (45.3)
Depressive	107 (62.2)	55 (57.3)	63 (62.4)	225 (61.0)
Bipolar	2 (1.2)	0 (0)	5 (5.0)	7 (1.9)
Somatic symptom and sleep-wake	5 (2.9)	8 (8.3)	12 (11.9)	25 (6.8)
Substance use	12 (7.0)	6 (6.3)	8 (7.9)	26 (7.0)
Feeding and eating	10 (5.8)	5 (5.2)	7 (6.9)	22 (6.0)
Obsessive-compulsive and related	12 (7.0)	7 (7.3)	5 (5.0)	24 (6.5)
Trauma- and stressor-related	7 (4.1)	8 (8.3)	12 (11.9)	27 (7.3)
Attention deficit/hyperactivity disorder	23 (13.4)	13 (13.5)	15 (14.9)	51 (13.8)
Other	1 (0.6)	2 (2.1)	1 (1.0)	4 (1.1)
Previous treatment				
Clinical admission	29 (16.9)	8 (8.3)	11 (10.9)	48 (13.0)
Day treatment	29 (16.9)	16 (16.7)	16 (15.8)	61 (16.5)
Outpatient	118 (68.6)	68 (70.8)	75 (74.3)	261 (70.7)
Total previous treatments, mean (SD)	2.49 (2.57)	2.39 (2.31)	2.46 (2.24)	2.46 (2.41)
Medication use‡	92 (53.5)	50 (52.1)	54 (53.5)	196 (53.1)
Antidepressants	66 (38.4)	40 (41.7)	39 (38.6)	145 (39.3)
Benzodiazepines	26 (15.1)	18 (18.8)	15 (14.9)	59 (16.0)
Antipsychotics	13 (7.6)	13 (13.5)	12 (11.9)	38 (10.3)
Mood stabilisers	5 (2.9)	8 (8.3)	5 (5.0)	18 (4.9)
Other psychopharmaceuticals	18 (10.5)	7 (7.3)	11 (10.9)	36 (9.8)

* Data denote number (percentage) of patients, unless otherwise specified.

† Based on the International Standard Classification of Education, 2011 version, range 0–7.

‡ Medication used ≥1 time in the past 4 months.

GST, group schema therapy; IST, individual schema therapy; sTAU, specialised treatment as usual.

**Table 2:** Comparison of time effects for the primary outcome between GST, IST and sTAU

Outcome, weeks, comparison	t	df	P value	d (95% CI)
Cluster-C PD severity				
Main time effect*				
34	−16.54	1161	<0.001	0.87 (0.76; 0.97)
51	−17.98	1161	<0.001	1.04 (0.92; 1.15)
104	−19.48	1161	<0.001	1.20 (1.08; 1.32)
34× treatment				
GST-IST	−0.23	1161	0.82	0.03 (-0.22; 0.28)
GST-sTAU	−1.39	1161	0.17	0.18 (-0.07; 0.43)
IST-sTAU	−1.03	1161	0.30	0.15 (-0.14; 0.43)
51× treatment				
GST-IST	0.57	1161	0.57	0.08 (-0.19; 0.35)
GST-sTAU	−2.35	1161	0.02	0.33 (0.05; 0.61)
IST-sTAU	−2.59	1161	0.010	0.41 (0.10; 0.72)
104× treatment				
GST-IST	0.73	1161	0.46	0.11 (-0.18; 0.39)
GST-sTAU	−0.78	1161	0.44	0.12 (-0.18; 0.42)
IST-sTAU	−1.33	1161	0.18	0.23 (-0.11; 0.56)

* The main time effect is the weighted mean across all three conditions.

GST, group schema therapy; IST, individual schema therapy; sTAU, specialised treatment as usual.

### Primary outcome

Estimated means of the primary outcome variable over time are presented in figure 2a. At post-booster, the decrease in cluster-C PD severity was significantly larger for GST and IST compared with sTAU. At 2 years, no significant differences between treatments were found. Sensitivity analyses in which a covariate was added to the cluster-C PD severity model, including type of cluster-C diagnosis, demographics, follow-up missingness and number of treatment sessions, did not change conclusions (online supplemental 7–10). When the model was re-estimated without outliers and influential cases, the GST-sTAU difference at post-booster lost significance (p=0.06; with an effect size shrinkage from 0.33 to 0.27, online supplemental sTable 7). All treatments showed significant symptom reduction over time with large (d>0.8) effect sizes (GST: d=1.20, IST: d=1.30 and sTAU: d=1.08).

**Figure 2:**
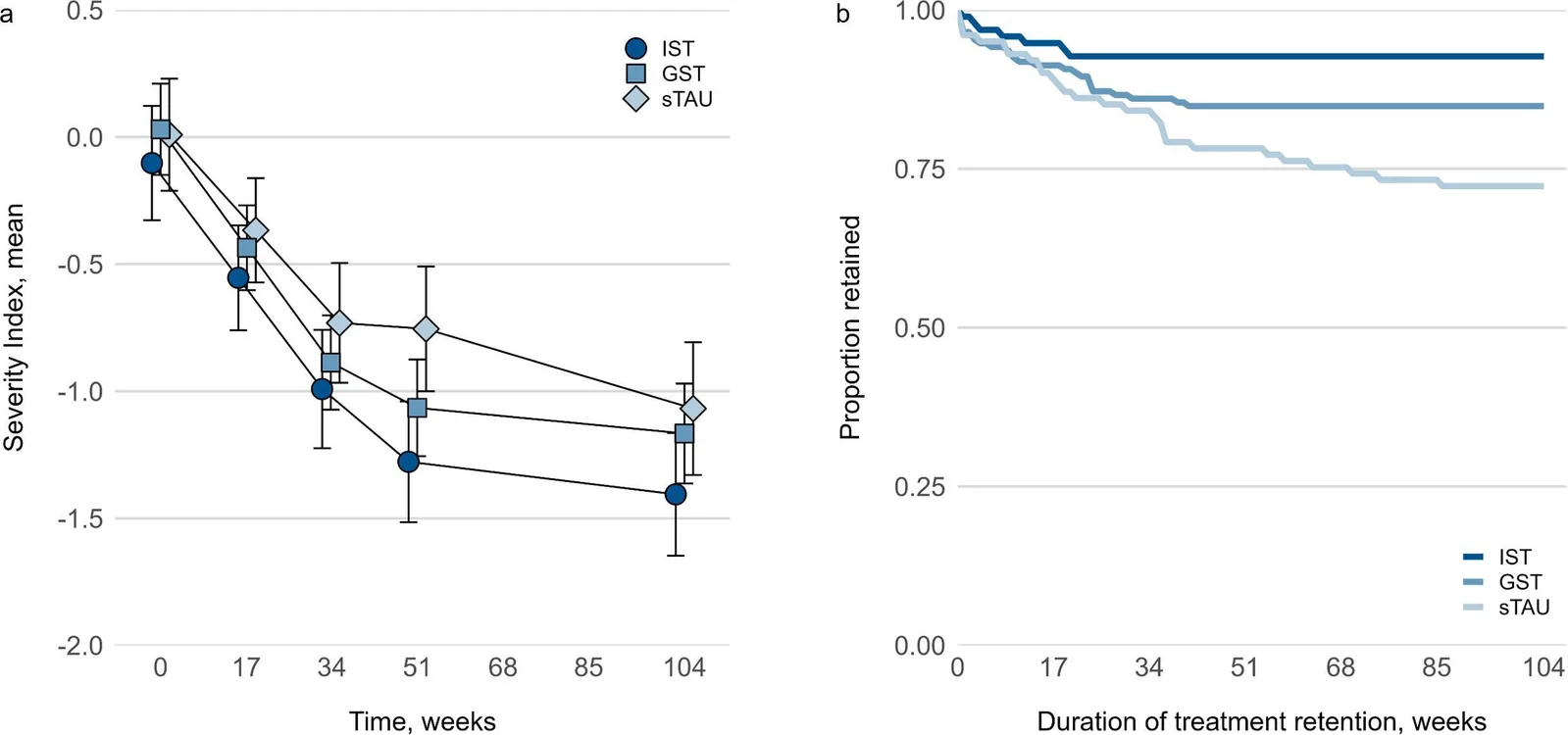
Change in primary outcome variable and duration of treatment retention. (**a**) Estimated marginal means and 95% CIs from the linear mixed model of the total cluster-C PD severity scores per treatment arm. (**b**) Treatment retention by weeks per treatment arm for 2 years. For treatment completers, treatment retention was the full observation period. GST, group schema therapy; IST, individual schema therapy; sTAU, specialised treatment as usual.

### Secondary outcomes

Estimated means of secondary outcome variables over time are presented in online supplemental sFigure 3. At 2 years, there was a significant improvement in all secondary outcomes across all treatments (effect sizes can be found in online supplemental sTable 4). Effect sizes of symptom decrease were large (d>0.8) for most outcome variables and treatments, with medium effect sizes (0.5≤d<0.8) for general quality of life (GST and sTAU), introversion (GST), dysfunctional schema modes (sTAU) and sleep problems (all treatments) and a small effect size (d<0.5) for IST and sTAU on introversion. When comparing treatments on symptom decrease at post-treatment, post-booster and 2 years after start of treatment, no significant differences were found on self-ideal discrepancy and introversion. For the other secondary outcomes, significant differences between treatments in symptom trajectories are described below (see online supplemental sTable 3 for an overview of all comparisons).

#### Differences between GST and IST

Only one difference was observed between GST and IST: for general quality of life, improvement at post-booster was significantly larger for IST than for GST, but no significant difference was observed at post-treatment or 2 years after the start of treatment.

#### Differences between GST and sTAU

At post-treatment, GST showed significantly more improvement than sTAU on psychopathology, general and mental health quality of life, happiness, PD-related beliefs, self-esteem, schemas, functional and dysfunctional schema modes and sleep problems. At post-booster, improvement was significantly larger for GST compared with sTAU for self-esteem and functional and dysfunctional schema modes. No significant differences were observed at 2 years after the start of treatment.

#### Differences between IST and sTAU

At post-treatment, IST showed significantly more improvement than sTAU on general functioning, psychopathology, general and mental health quality of life, happiness, PD-related beliefs, self-esteem, schemas, functional and dysfunctional schema modes and sleep problems. At post-booster, IST showed significantly more improvement than sTAU on psychopathology, general and mental health quality of life, happiness, PD-related beliefs, self-esteem, schemas and functional and dysfunctional schema modes. Finally, the only difference observed at 2 years was for functional and dysfunctional schema modes, with IST superior to sTAU. When the models were re-estimated without outliers and influential cases, the difference between IST and sTAU at post-booster lost significance for happiness (p=0.06; with an effect size shrinkage from 0.36 to 0.35, online supplemental sTable 8).

### Treatment retention

Treatment retention is shown in figure 2b. On average, GST treatment lasted 340.40 days (SD 9.982, 95% CI 320.69 to 360.10), IST lasted 389.19 days (SD 13.590, 95% CI 362.21 to 416.17), and sTAU lasted 418.23 days (SD 21.444, 95% CI 375.68 to 460.77); see online supplemental sTable 6 for the average amount of sessions per treatment arm at each assessment. At 1 year after start of treatment, treatment retention was 84.9% for GST, 92.7% for IST and 78.2% for sTAU. According to the study protocol, GST and IST were scheduled to end 1 year after treatment initiation; hence, retention rates remained the same. For sTAU, however, treatment retention lowered to 72.3% at 2 years after the start of treatment. Kaplan-Meier survival analysis on treatment retention at 1 year showed no significant differences between GST and IST ((1)²=3.344, p=0.07) and GST and sTAU (χ(1)²=1.779, p=0.18), but did show a significant difference between IST and sTAU (χ(1)²=7.816, p=0.005). At 2 years, significant differences were found between GST and sTAU ((1)²=5.645, p=0.02) and IST and sTAU (χ(1)²=13.239, p<0.001).

### Treatment satisfaction

Satisfaction with the allocated treatment was assessed at 2 years after start of treatment and analysed with a GLMM with a log link and random intercept for treatment site. On average, participants in all three conditions were satisfied with the treatment they received (estimated means in original scale, GST: M=1.84, SD=0.07; IST: M=1.56, SD=0.07; sTAU: M=1.84, SD=0.10). There was a significant difference in treatment satisfaction between GST and IST (t=2.82, df=249, p=0.005, d=0.21, 95% CI 0.06 to 0.36) and IST and sTAU (t=−2.29, df=249, p=0.02, d=0.21, 95% CI 0.03 to 0.40) with higher treatment satisfaction in IST, and no significant difference between GST and sTAU (t=−0.01, df=249, p=0.99, d=0.00, 95% CI −0.18 to 0.18).

### Adverse events

One suicide, considered not treatment-related, was reported in GST at 6 months.

## Discussion

This RCT compared the effectiveness of GST, IST and sTAU as treatments of cluster-C PDs. For the primary outcome, cluster-C PD severity, GST and IST were superior to sTAU after 1 year of treatment but did not differ at 2 years after the start of treatment. GST was superior to sTAU for most secondary outcome variables at 34 weeks and for a few outcome variables at 1 year, whereas IST was superior to sTAU on most outcome variables at both 34 weeks and 1 year. Despite a pattern where IST appeared to outperform GST on all outcome variables, only one difference was significant at 1 year (quality of life). No differences were found between treatments on any of the outcome variables at 2-year follow-up except on functional and dysfunctional schema modes, where IST was superior to sTAU, and on treatment retention, where both GST and IST had fewer treatment dropouts than sTAU. Effect sizes of symptom decrease at 2 years were large for most outcome variables and treatment arms.

Compared to previous research, effect sizes resembled those found in the GST pilot study,^15^ and those found for IST, TAU and COP in a trial by Bamelis and colleagues with (mainly) patients with cluster-C PD.^10^ Dropout was 15.1% in GST, 7.3% in IST and 27.7% in sTAU in the current investigation, compared with 11.7% in the GST pilot study, and 10.6% in IST after 1 year (corrected for influence of cohort), 37.8% in TAU and 22% in COP after 3 years in the Bamelis trial, suggesting that sTAU dropout rates differed most with previous research. Similarly, they found that IST was more effective regarding PD recovery than COP and sTAU, whereas almost no differences between treatments were found at 2-year follow-up in the current study. Sensitivity analyses indicated that for cluster-C PD severity, this was not related to different symptom trajectories of sTAU patients with and without a follow-up assessment (online supplement 9). Nevertheless, study dropout for sTAU at year 2 was considerable, which may have influenced follow-up results. Although sTAU often lasted longer and patients received more treatment sessions in the second year compared with GST and IST, controlling for additional sessions did not change results (online supplement 10). As treatment dose in sTAU was not randomised but determined per patient, it is still possible sTAU effects approached those of the ST arms due to treatment many sTAU participants received in year 2, and it remains to be studied how this impacts the cost-effectiveness of sTAU relative to GST and IST. An economic evaluation is currently underway and will be reported in a separate article. A potential explanation for differences with the Bamelis trial could lie in treatment options for cluster-C PD evolving since then, such as two of the most commonly received sTAU treatments in the present study: GITPD and SCB.^25 26^ Therefore, the comparable long-term effectiveness of sTAU and ST in the current study may be attributed to the improvement of treatments for cluster-C PDs.

However, ST formats yielded earlier improvements on most outcomes and greater acceptability (eg, lower dropout) than sTAU. Given that the symptoms experienced by patients with PDs are persistent and severely impair daily life, it is highly desirable that symptoms are alleviated faster. Potential explanations for the favourable effects of ST may be derived from patients’ experiences with the ST protocols, which were explored qualitatively (see the detailed results of this substudy in the dedicated manuscript^27^). Interviews indicated that patients experienced protocol aspects and techniques in ST as beneficial for recovery.^27^ They appreciated the therapeutic relationship (ie, limited reparenting), and although techniques were sometimes difficult or confrontational, they were perceived as inducing change. Within GST and group formats of sTAU (but not individual formats), patients indicated a need for more (individual) sessions to work on their personal recovery goals, which may explain why IST outperformed sTAU more often than GST. Nonetheless, group therapy offered unique learning experiences that were highly valued by patients. Future research could study the effects of combined individual and group ST, which was superior to predominantly GST in borderline PD.^17^ Furthermore, the fact that patients in the ST group showed improvement more quickly than those in the sTAU group may have been influenced by differences in the timing of the assessments: in the ST arms, these were timed to align with the treatment stages, whereas this was not the case for sTAU. As sTAU treatment consisted of longer, consecutive treatments which, in some cases, continued beyond the 1-year and 2-year assessments, the assessment took place at a different stage of the treatment process. Subsequent improvements in these patients may not have been fully captured, and patients may have assessed their symptoms differently, knowing that their treatment had not yet been completed.

Despite possible advantages and positive experiences of GST, not all patients might benefit equally from GST. The preceding GST pilot study^15^ found indications for a reduced treatment effect for OCPD compared with APD (medium and large effect sizes, respectively). Interestingly, the current study found large effect sizes for both diagnoses within GST (online supplement 7). This may be attributed to changes in the GST protocol,^20^ which now includes components explicitly aimed at addressing OCPD-specific schema modes. A follow-up study will explore which patient characteristics predict differential effectiveness of IST and GST.

This study has several limitations that warrant further discussion. First, part of the data collection took place during the COVID pandemic, creating an atypical format with online sessions. Second, the frequency and burden of the assessments led some of the participants to terminate participation in the study and, for some, to dropout of treatment (see figure 1). These dropouts can compromise the validity of the findings. Third, the distribution of diagnoses within the cluster-C PD group was uneven, with fewer patients with dependent and obsessive-compulsive PD than was expected based on prevalence numbers.^28^ Results are less generalisable to these subgroups. Fourth, although inclusion and exclusion criteria were limited to a minimum, aiming for high external validity, for practical reasons we did not include ‘other specified’ diagnoses, and for safety reasons, we excluded patients with acute suicidality. These patients are seen regularly in clinical practice and further research is therefore warranted. Fifth, our interpretation of treatment effects of sTAU compared with the ST arms at follow-up in light of additional care received after treatment is limited, on the one hand, due to lacking information on the type of additional care received after the study treatment (see online supplement 10), and on the other hand, due to the difference between additional and ongoing treatment being inherently difficult to determine in the sTAU arm, as treatment itself often consisted of stacking different treatments ‘as needed’. Lastly, ST could have had an advantage over sTAU due to centrally organised training and supervision, which may have led to group differences in study commitment. Additionally, since sTAU included a variety of different specialised psychotherapies, it remains to be studied how ST relates to specific forms of interventions, which is currently explored in two other RCTs.^29 30^

## Clinical implications

In conclusion, this multicentre RCT showed IST and GST to be more effective than sTAU after 1 year of treatment, with no differences at follow-up. Combined with higher retention rates, our results suggest individual and group ST to be a good intervention for cluster-C PD in clinical practice. The results of the studies on cost-effectiveness and predictors of differential effectiveness will further substantiate choices in treatment selection and allocation.

## Supplementary material

10.1136/bmjment-2026-302929 online supplemental file 1

## Data Availability

Data are available upon reasonable request.
