# Supplementary material for: Quest for optimal treatment of cluster-C personality disorders: evaluating schema therapy formats in a multicentre randomised controlled trial
Source: BMJ Ment Health. 2026 Oct 26;29(1):e302929. doi: 10.1136/bmjment-2026-302929 (PMC13711618; doi:10.1136/bmjment-2026-302929)
Supplement: Supplementary file 1 [file bmjment-29-1-s001.pdf]

Supplemental Material

Supplement 1. Deviations from the original plan
sTable 1. Overview of Specialized Treatment as Usual (sTAU) Treatments per Site
Supplement 2. Treatment Integrity
sTable 2. Treatment Integrity for Group and Individual Therapy Formats
Supplement 3. Outcome measures
Supplement 4. Selection of Random Effects and Covariance Structure for (G)LMM analyses
Supplement 5. Effect size calculations
sTable 3. Comparison of Time Effects for Secondary Outcomes Between GST, IST and TAU
sTable 4. Estimated Means, 95%CIs, and Effect Sizes for the Primary and Secondary Outcome Variables
Supplement 6. Reliable Change Index for Cluster-C PD Severity
Supplement 7. Analyses with Cluster-C Diagnosis
sTable 5. Sensitivity analysis with cluster-C diagnosis added to the Severity Index LMM
sFigure1. Change in Severity Index Scores per Diagnosis within Treatment
sFigure 2. Change in Severity Index Scores per Treatment within Diagnosis
Supplement 8. Sensitivity Analysis with Demographic Variables
Supplement 9. Sensitivity Analysis with Missingness at Follow-up
Supplement 10. Sensitivity Analyses with Treatment Sessions
sTable 6. Number of Allocated Treatment Sessions per Assessment
sTable 7. Sensitivity Analysis Results Cluster-C PD Severity without Outliers and Influential Cases
sTable 8. Sensitivity Analysis Results Happiness without Outliers and Influential Cases
sFigure 3. Change in Secondary Outcome Variables
sReferences

Supplement 1. Deviations from the original plan

Sites

Due to the closure of PsyQ Amsterdam, most patients in treatment were transferred to a new site: the Academic Centre for Trauma and Personality (ACTP) in Amsterdam. Three patients transferred along with their therapists to a new location (PsyQ Zaandam and PsyQ Utrecht).

Patient Recruitment & Sample Size

In the original study protocol, we aimed to include 378 patients (162 GST, 108 IST, 108 sTAU) in the study. However, due to the termination of one participating site and additional difficulties within other sites to include the planned number of patients, we decided to stop inclusion at N = 375 to prevent further delay of the study. A second power-analysis showed that with 375 participants, with an expected effect size of *d* = .44 resulted in a power of .898, indicating only minor loss in power for the adjusted sample size.

Due to lower influx of patients with a cluster-C PD than expected some of the sites were not able to reach the 42 patients. To compensate, PsyQ Amsterdam/ACTP, PsyQ Rotterdam, IPGGZ Veendam, and GGZ-OB Oss ran an extra cohort, and GGZ-OB Helmond ran two extra cohorts.

Randomisation

Due to fluctuations in participant recruitment, some randomisations were performed with different ratios but always ensuring that participants could be assigned to one of the three arms. Consequently, as well as due to altered capacity at the sites, the distribution among the therapy arms deviated from the original planning of 162 GST, 108 IST and 108 sTAU.

Group sizes of GST

Within GST, groups generally consisted of 5 to 9 patients; however, in rare cases, temporary deviations occurred due to difficulties with patient inflow wherein group sizes were reduced to 2 to 3 patients.

Statistical plan

The trial was explicitly designed and powered as a superiority study comparing GST to TAU and IST, and our primary analyses therefore target differences in effectiveness. Some phrasing in the protocol paper may suggest a non-inferiority perspective, but the study’s hypotheses and analytic strategy are grounded in a superiority design.

For the drop-out analysis, a GLMM survival analysis with site as random effect was planned. However, adding a random intercept for site led to a non–positive definite Hessian matrix, indicating an unstable fit and unreliable estimates of fixed effects. Without the possibility of nesting, a Kaplan-Meier survival analysis was the chosen alternative for analyzing treatment retention.

Primary and secondary analyses were blinded for treatment type by IG and analyzed by ASV. Unequal effect sizes did make it possible to deduce which of the three groups represented the GST condition e.g. from residual plots, but blinding in this way was considered to still reduce bias regarding IST/sTAU.

sTable 1. Overview of Specialized Treatment as Usual (sTAU) Treatments per Site

Site	SCBT - group	SCBT- individual	CBT - individual	Psychodynamic psychotherapy - group	Psychodynamic psychotherapy - individual	APT - individual	MBT - individual	GIT-PD group or individual	Structuring and supportive conversations	Combination of Therapies*	Drop-out before start	**Total**
PsyQ Amsterdam/ ACTP	4					8						**12**
GGZ-OB Boxmeer								5				**5**
Emergis			2							6		**8**
IPGGZ Groningen	2		2							8	2	**13**
GGZ-OB Helmond								13				**13**
GGZ-OB Oss			1					9	1			**11**
PsyQ Rotterdam				9	2							**11**
PsyQ Utrecht			3			1			1	2		**7**
IPGGZ Veendam	3	6								4		**13**
PsyQ Zaandam						1	5			2		**8**
**Total**	**8**	**6**	**7**	**9**	**2**	**10**	**5**	**27**	**2**	**22**	**2**	**101**

Abbreviations: sTAU = Specialized Treatment as Usual; SCBT = Short-term Schema-based Cognitive Behavioral therapy; CBT = Cognitive Behavioral Therapy; EMDR = Eye Movement Desensitization and Reprocessing therapy; APT = Affect Phobia Therapy; MBT = Mentalization Based Treatment; GIT-PD = Guideline-Informed Treatment for Personality Disorders.

***Combination of Therapies consisted of (*n* = 22)**

- CBT & EMDR (*n* = 3)
- CBT & SCBT & PMT & Structuring and supportive conversations (*n* = 2)
- CBT & SCBT (individual or group) (*n* = 4)
- CBT & SCBT & PMT (*n* = 1)
- CBT & SCBT & EMDR (*n* = 2)
- CBT & Mindfulness & RET & EMDR (*n* = 1)
- CBT & Skills training (*n* = 1)
- CBT & MBT & individual psychotherapy (*n* = 1)
- CBT & training negative self-image (according to Manja de Neef's method) & EMDR (*n* = 1)
- SCBT & structuring and supportive conversations (*n* = 2)
- SCBT & PMT & structuring and supportive conversations (*n* = 1)
- APT & MBT (*n* = 1)
- Psychodynamic psychotherapy & PEP4all (*n* = 1)
- Individual psychotherapy with SFT and MBT elements (*n* = 1)

Supplement 2. Treatment Integrity

Therapy sessions were video recorded to assess treatment adherence (i.e. whether the therapy protocol was followed as instructed) and competence (i.e. the quality of the techniques performed). All raters were blind for therapy condition, followed training, and participated in calibration sessions with each other and the first authors. Treatment integrity was determined in two rounds; first GST-sessions were compared to sessions of group treatments in the sTAU-arm (gsTAU) and second, IST-sessions to sessions of individual treatment in the sTAU-arm (isTAU). Recordings were randomly selected from different phases of the therapy for every therapy arm. Interrater agreement (ICC) between the raters was determined by the guidelines of Koo et al.[1](i.e.: two-way random effects, average measures, absolute agreement). As we observed systematic differences between raters, we corrected the ratings by subtracting each rater's weighted mean across the treatment arms (see sTable 2).

Group sessions

A total of 179 recordings were selected: 125 GST and 54 for gsTAU. Eleven master-level students and two health psychologists rated the recordings. Recordings were scored on a self-composed rating scale, derived from items of the Group Schema Rating Scale-Revised [2,3] and the Schema Therapist Competency Scale for Individual Therapy Sessions (STCS-I-1)[4] to determine adherence and competence. Adherence consisted of items indicating the 0 (absence) or 1 (presence) of GST-techniques, while competency was rated on how well these techniques were performed, ranging from 1 (very poorly) to 6 (excellent).

Interrater agreement was moderate (.52) for adherence to the protocol and low (.13) for competence. However, GST-sessions were still rated significantly higher than gsTAU-sessions on GST-adherence and competence (see Table 1). A specific comparison was conducted between the GST sessions and the SCBT-g (part of the sTAU arm) to ensure that the two interventions were sufficiently distinct from one another. T-tests showed that the GST sessions contained more schema therapeutic techniques (e.g. adherence) compared to the SCBT-g sessions, $t(152) = 3.04$, $p = .003$, $d = 0.64$, but no significant differences in competence of the observed techniques were found, $t(152) = 1.0$, $p = .27$, $d = 0.23$.

Finally, the facilitative conditions and explicit directiveness scales of the Collaborative Study Psychotherapy Rating Scale, version 6[5,6] were rated to assess general therapeutic style: The facilitative conditions scale (nine items), and the explicit directiveness scale (three items) both ranging from 1 (not or very little) to 7 (extensively or often). Because facilitative conditions and explicit directiveness were rated in GST, IST and gsTAU and isTAU, the interrater agreement was determined based on all recordings and showed to be low (.26-.45). Nonetheless, facilitative conditions and explicit directiveness were, as expected, rated higher in GST than gsTAU (sTable 2).

Individual sessions

A total of 191 recordings were rated by nine master students: 161 IST and 30 isTAU. First, the adherence of the protocol was assessed by indicating if a list of 43 ST techniques[6] were absent (0) or present (1). In addition, the raters indicated on a 7-point Likert scale how often these schema techniques were applied, ranging from 1 (not or very little) to 7 (extensively or often). Competence was assessed using the Schema Therapist Competency Scale for Individual Therapy Sessions (STCS-I-1;)[4] consisting of 14 schema therapeutic techniques that are rated on scale ranging from 0 (very poor) to 6 (excellent). Finally, the facilitative conditions and explicit directiveness scales[5] were also assessed for the individual interventions.

ICC between the raters was moderate (.64) for competence, and good for adherence (.79 (absent/present) and .79 (scale)). As expected, we found significant differences in the adherence and competence of performed ST techniques, as well as higher facilitative conditions and explicit directiveness in favour of IST in comparison to isTAU. (see sTable 2).

Discussion

Although it was primarily important for this study to demonstrate that the treatments differed in terms of the degree of schema therapy techniques used, it is striking that the ICC between raters was low, particularly for the group therapy sessions. This therefore requires further discussion. After observing the low ICC in the first round (group therapy), the training was intensified, and more calibration sessions were added for the second round (individual therapy). Indeed, the ICC was of better quality in the second round than in the first. The only consistent outcomes between the two rounds were the FC and ED scales. For these, we see that the second round has a higher ICC (for FC .09 in the group round versus .63 in the individual round, and for ED .11 versus .41). However, because different

recordings and raters were used in both rounds, no causal conclusions can be drawn about het effects of the intensified training. Other possibilities include that group sessions and group rating scales are more challenging to score than individual sessions and rating scales. Additionally, previous research suggests that students without clinical experience may lack the detailed knowledge of treatment components needed to adequately assess treatment competency.[7]

sTable 2. Treatment Integrity for Group and Individual Therapy Formats

	Original Mean		Corrected Mean[a]		Test-statistic
**Group therapy**	***GST (n=125)***	***gsTAU (n=54))***	***GST (n=125)***	***gsTAU (n=54))***	
GST Adherence (absent/present)	0.71 (0.13)	0.54 (0.17)	0.12 (0.13)	-0.05 (0.16)	*t* (82.21) = 6.80, ***p* <.001**, *d* =1.22
GST Competence	4.24 (0.51)	4.13 (0.72)	0.23 (0.42)	-0.15 (0.64)	*t* (73.11) = 4.02, ***p* <.001**, *d* = 0.77
Facilitative Conditions	5.12 (0.47)	4.88 (0.61)	0.22 (0.43)	-0.11 (0.56)	*t* (81.15) = 3.88, ***p* <.001**, *d* = 0.70
Explicit Directiveness	4.59 (0.54)	4.71 (0.59)	0.17 (0.48)	-0.03 (0.45)	*t* (177) = 2.66, ***p* = .009**, *d* = 0.43
**Individual therapy**	***IST (n=161)***	***isTAU (n=30)***	***IST (n=161)***	***isTAU (n=30)***	
IST Adherence (absent/present)	0.43 (0.11)	0.24 (0.09)	0.09 (0.08)	-0.10 (0.06)	*t* (50.59) = 15.05, ***p* <.001**, *d* = 2.46
IST Adherence (scale)	2.25 (0.36)	1.51 (0.26)	0.36 (0.36)	-0.38 (0.23)	*t* (58.21) = 14.39, ***p* <.001**, *d* = 2.15
IST competence	4.09 (0.67)	3.07 (0.68)	0.53 (0.64)	-0.53 (0.61)	*t* (189) = 8.48, ***p* <.001**, *d* = 1.69
Facilitative Conditions	4.70 (0.70)	4.36 (0.74)	0.20 (0.65)	-0.22 (0.72)	*t* (189) = 3.21, ***p* =.002**, *d* = 0.64
Explicit Directiveness	4.16 (0.48)	3.79 (0.61)	0.25 (0.38)	-0.24 (0.51)	*t* (35.37) = 5.03, ***p* <.001**, *d* = 1.21

Abbreviations: GST = Group Schema Therapy, IST = Individual Schema Therapy, isTAU = individual specialized treatment as usual, gsTAU = group specialized treatment as usual.
[a]As systematic differences between raters were observed, ratings were corrected by subtracting each rater's weighted mean across the treatment arms (i.e. 'corrected mean').

Supplement 3. Outcome measures

All interviews and questionnaires were administered to or completed by the patients themselves. The only exception was the reporting of serious adverse events, which were documented by the treating therapist.

Primary Outcome

Primary outcome was severity of the primarily diagnosed personality disorder, measured using diagnosis-specific interviews: Avoidant Personality Disorder Severity Index (AVPDSI)[8], Obsessive-Compulsive Personality Disorder Severity Index (OCPDSI)[9], and Dependent Personality Disorder Severity Index (DPDSI)[10].The AVPDSI contains seven subscales aiming to measure the seven PD-criteria of the DSM-5 for the avoidant PD. A total of 53 item-pairs assesses the avoidance and experienced fear over the past month, rated on a scale from 0 (never) to 5 (every day). The OCPDSI and DPDSI both contain eight subscales, corresponding to the eight DSM-criteria of the obsessive-compulsive PD and dependent PD. The OCPDSI consists of 51 questions, whereas the DPDSI contains 47 items, all scored on a scale from 0 (never) to 5 (every day). In addition, both interviews include two questions at the end of each subscale assessing the burden and impact of the subsection symptoms on a scale from 1 (not at all) to 10 (unbearably much). The AVPDSI has demonstrated to be a valid and reliable instrument, with a good sensitivity for change[8] Reliability was high for all time points in the current study (AVPDSI: α = .94-.96, DPDSI: α = .96-.97, and OCPDSI: α = .92-.96). Interrater agreement ranged from good to excellent (AVPDSI: .85, DPDSI: .98, and OCPDSI: .92).

Secondary outcome

Functioning

The World Health Organization Disability Assessment Schedule II (WHODAS-II)[11] was conducted, addressing six domains of functional impairment (i.e. cognition, mobility, self-care, getting along, life activities and participation). The WHODAS consists of 36 items measured on a 5-point Likert scale, ranging from 0 (none) to 4 (extreme/cannot do). The total score using the complex scoring method was used as an outcome variable.[12] The psychometric properties of the WHODAS II have demonstrated to be satisfactory.[13] Cronbach's alpha ranged from .88 to .90 for the different time points. Interrater agreement was excellent (.95).

General psychopathology

The Brief Symptom Inventory (BSI)[14] was conducted to measure general psychopathology using nine subscales: Somatization, obsession-compulsion, interpersonal sensitivity, depression, anxiety, hostility, phobic anxiety, paranoia and psychoticism. The BSI consists of a total of 53 items, scores on a 5-point Likert scale ranging from 0 (not at all) to 4 (extremely). A total global severity index score was used in analyses. The BSI exhibits satisfactory psychometric properties.[15] Reliabilities were high for all time points (α =.95 - .97).

Quality of Life (QoL)

Two QoL-measurements were administered: The long-established EQ-5D-5L[16] and the Mental Health Quality of Life Questionnaire (MhQol-7D)[17], specifically designed to measure QoL in populations with mental illness. The EQ-5D-5L contains five items addresses different health state dimensions (i.e. mobility, self-care, usual activities, pain/discomfort and anxiety/depression). The severity of each dimension can be scores on five levels ranging from 1 (no problems) to 5 (extreme problems/ unable to). The Dutch tariffs were used to calculate an index score.[18] The EQ-5D-5L has satisfactory psychometric properties across different populations[19], although Cronbach's alpha ranged from .66 to .76 in the current study.

The MHQoL-7D addresses seven life domains (i.e. self-image, independence, mood, relationships, daily activities, physical health and future), which can be answered a 4-point scale ranging from 0 (very dissatisfied) to 3 (very satisfied). An index score was obtained using the Dutch tariffs.[20] The MHQoL-7D has a high internal consistency and adequate validity.[17] Reliability on the different timepoints ranged from acceptable to good (α = .75-.87).

Happiness

Happiness was measured with a single question.[21] Participants were asked to indicate their level of happiness over the past months on a 7-point Likert scale ranging from 1 (completely happy) to 7 (completely unhappy). The psychometric properties of this question are shown to be sound.[21,22]

Personality Disorder beliefs
The Personality Disorder Beliefs Questionnaire (PDBQ)[23,24] was used to assess PD-related beliefs. In this study, only the 32 items were used that assess beliefs of APD (10 items), DPD (13 items) and OCPD (9 items). The strength of belief in each item was rated using a 100 mm Visual Analogue Scale (VAS), ranging from 0 (I don't believe this at all) to 100 (I believe this completely). The subscales of the PDBQ showed satisfactory validity and reliability.[24] Reliabilities of the three PDBQ scales were good to excellent at the different timepoints (Avoidant: α = .90 - .95, Dependent: α = .91 - .94, and Obsessive-compulsive: α = .79-.88).

Self-esteem
Self-esteem was conducted with the Rosenberg's Self-Esteem Scale (RSES).[25] The RSES consists of 10 items rated on a 4-point Likert scale ranging from 0 (strongly agree) to 3 (strongly disagree). The reliability and validity of the Dutch RSES are well established.[26] Cronbach's alpha's ranged from .97 to .93 for the different time points.

Self-ideal discrepancy
The amount of self-ideal discrepancy was assessed using the Miskimins Self-Goal-Other Discrepancy Scale (MSGO).[27] The MSGO contains 15 item-pairs that assess constructs in two ways: Once 'as I am now' (current self) and once 'as I would like to be' (goal). The items can be answered on a 100 mm VAS, in which 0 represents the counterpart of 100; for example, 0 (socially adept) and 100 (socially awkward). The total discrepancy between the current self- and goal-items is then calculated and used as a measure of self-discrepancy. In multiple studies, the MSGO demonstrates to be satisfactory on validity and reliability measures.[28–30] Reliability ranged from .83 to .90 for the different time points.

Introversion
Levels of introversion were measured by the Introversion Inventory.[31] This questionnaire was developed as part of the QUEST-CLC study, with the aim of creating a brief, open-access instrument specifically designed to assess introversion—a personality trait particularly relevant in individuals with a cluster-C PD. The Introversion Inventory consists of 19 items, assessed on a 5-point Likert scale ranging from 0 (not true at all) to 4 (completely true). The introversion Inventory showed good psychometric properties in the sample of cluster-C patients and a control group.[32] Cronbach's alpha varied from .91 to .92 in our sample.

Maladaptive Schemas
To assess maladaptive schemas, the Young Schema Questionnaire-Revised (YSQ-R)[33,34] was conducted. The YSQ-R contains 90 items assessing 18 schemas. The total score represents the number of maladaptive schemas within. All items are assessed on a 6-point Likert scale ranging from 1 (completely untrue of me) to 6 (describes me perfectly). The YSQ-R exhibits excellent psychometric properties.[33] Cronbach's alpha ranged from .97 to .99 in the current study.

Schema modes
The presence of schema modes was measured using the Schema Mode Inventory-2 (SMI-2).[35,36]The SMI-2 aims to measure 17 dysfunctional and one functional schema mode (the healthy adult mode) using 192 items, scored on a 6-point Likert scale ranging from 1 (never or hardly ever) to 6 (always). Two outcome variables were utilized: a total score representing dysfunctional schema modes and a total score representing the healthy adult mode. Together, these scores were used to assess overall schema mode functioning. The SMI-2 has good psychometric properties.[35] Reliability was high at the different timepoints (α = .97 - .99).

Sleep Problems
The SLEEP-50 was conducted to measure sleep quality over the previous weeks[37]. The SLEEP-50 assesses nine domains (i.e. Sleep apnea, insomnia, narcolepsy, restless legs/periodic limb movement disorder, circadian rhythm sleep disorder, sleepwalking, nightmares, factors influencing sleep and impact of sleep complaints on daily functioning). The questionnaire contains 50 items rated on a 4-point scale ranging from 0 (not) to 4 (a lot). The psychometric properties of the SLEEP-50 are sound.[37] In the current study, reliability ranged from .87 to .92.

Serious Adverse Events

Serious Adverse Events (SAE) comprised events that result in life-threatening situation, require hospitalization, or results in significant disability or dead. In such cases, the SAE is reported within 24 hours to the principal investigators and the relatedness to the intervention will be studied. Findings of this investigation will be reported to the ethical committee of the University of Amsterdam.

Treatment satisfaction
At follow-up, participants were asked whether they were satisfied with the treatment they followed in the context of the study on a 5-point scale ranging from 1 (completely satisfied) to 5 (not at all satisfied).

Supplement 4. Selection of Random Effects and Covariance Structure for (G)LMM analyses

For normally distributed data (with restricted maximum likelihood as the estimation method), a random intercept for participant and treatment site was added if it resulted in a significant improvement of the model fit, determined based on the Aikake information criterion (AIC) and Bayesian information criterion (BIC). The addition of a random intercept for inclusion cohort did not result in an improvement of model fit or even worsened the model fit. The addition of any random slope either did not result in an improvement of model fit and/or the fitting algorithm could not be made to converge without problems. Similarly, an autoregressive of order 1 (AR1) covariance structure was used to model the correlation pattern of the repeated assessments, as other covariance structures (unstructured, ARMA11, ARMA02, (heterogeneous) toeplitz, (heterogeneous) compound symmetry) resulted in an inferior fit, model instability, or non-convergence.

For GLMMs (with residual pseudo-likelihood as the estimation method), the selection of random effects and covariance structure could not be based on AIC and BIC comparisons as these are based on pseudo-likelihoods[38]. Therefore, these decisions were made based on LMMs applied to data transformed with a Box-Cox transformation, after which the resulting random components were added to the final GLMM model (analyzed in SPSS (version 29) instead of R (version 4.3.2), as R does not support residual pseudo-likelihood).

Overview of random effects for all outcome variables:

Severity index. Random intercept for participant and treatment site.
MSGO. Random intercept for participant.
Introversion inventory. Random intercept for participant and treatment site.
WHODAS-II. Random intercept for participant and treatment site.
SLEEP-50. Random intercept for participant and treatment site.
Happiness. Random intercept for participant.
PDBQ. Random intercept for participant and treatment site.
BSI. Random intercept for participant and treatment site.
YSQ-R. Random intercept for participant and treatment site.
MHQoL-7D. Random intercept for participant and treatment site.
EQ-5D-5L. Random intercept for participant and treatment site.
RSES. Random intercept for participant and treatment site.
SMI-2 functional. Random intercept for participant.
SMI-2 dysfunctional. Random intercept for participant and treatment site.

Supplement 5. Effect size calculations

For LMM analyses, Cohen's d and accompanying 95% CI's for the difference in change between treatments was calculated by multiplying the coefficient and their lower and upper bounds by the time over which the estimation was done, and dividing it by the standard deviation of the baseline scores.[39] For GLMM analyses, the standard deviation of the baseline scores was obtained from a GLMM on the baseline data with only a fixed intercept, so that both the coefficients and baseline SD would be in the transformed scale.

Supplement 6. Reliable Change Index for Cluster-C PD Severity

Reliable change[40], consisting of reliable improvement as well as reliable deterioration, on the cluster-C PD severity index was estimated for all patients that completed the follow-up assessment. Reliable change was calculated by subtracting a person's baseline score from their follow-up score on their respective PD severity index (avoidant, obsessive-compulsive, or dependent) and dividing it by the standard error of difference between these scores $\sqrt{(2SE^2)}$, *where* $SE = S1\sqrt{(1\text{-}rxx)}$, S1 = the baseline standard deviation, and rxx = the test-retest reliability. A person was deemed "reliably improved" when their reliable change was equal to or over 1.96 and deemed "reliably deteriorated" when their reliable change was equal to or under-1.96. Within GST (N = 122), 69.67% of patients showed reliable improvement while 6.56% deteriorated. Within IST (N = 76), 71.05% of patients showed reliable improvement while 2.63% deteriorated. Within TAU (N = 57), 66.67% of patients showed reliable improvement while 7.02% deteriorated.

sTable 3. Comparison of Time Effects for Secondary Outcomes Between GST, IST and TAU

**Outcome, weeks, comparison**	***t***	***df***	***P***	***d* (95%CI)**
General functioning				
Main time effect[a]				
34	-13.14	1146	<0.001	0.66 (0.56; 0.76)
51	-15.22	1146	<0.001	0.84 (0.74; 0.95)
104	-14.17	1146	<0.001	0.84 (0.73; 0.96)
34 x treatment				
GST-IST	-0.20	1146	0.84	0.02 (-0.21; 0.26)
GST-sTAU	-1.82	1146	0.07	0.22 (-0.02; 0.46)
IST-sTAU	-1.43	1146	0.15	0.20 (-0.07; 0.47)
51 x treatment				
GST-IST	0.88	1146	0.38	0.12 (-0.14; 0.38)
GST-sTAU	-1.67	1146	0.10	0.23 (-0.04; 0.49)
IST-sTAU	-2.26	1146	0.02	0.34 (0.05; 0.64)
104 x treatment				
GST-IST	0.00	1146	0.10	0.00 (-0.28; 0.28)
GST-sTAU	-0.41	1146	0.68	0.06 (-0.23; 0.35)
IST-sTAU	-0.37	1146	0.71	0.06 (-0.26; 0.39)
Psychopathology[b]				
Main time effect[a]				
34	11.82	997	<0.001	0.65 (0.54; 0.76)
51	12.90	595	<0.001	0.79 (0.67; 0.91)
104	12.51	396	<0.001	0.84 (0.71; 0.97)
34 x treatment				
GST-IST	-1.29	979	0.20	0.19 (-0.10; 0.49)
GST-sTAU	-2.68	1009	0.007	0.41 (0.11; 0.71)
IST-sTAU	-3.50	1002	<0.001	0.60 (0.27; 0.94)
51 x treatment				
GST-IST	-1.12	577	0.26	0.19 (-0.14; 0.52)
GST-sTAU	-1.56	608	0.12	0.27 (-0.07; 0.62)
IST-sTAU	-2.36	599	0.02	0.46 (0.08; 0.85)
104 x treatment				
GST-IST	-0.22	374	0.82	0.04 (-0.32; 0.40)
GST-sTAU	-0.75	414	0.45	0.14 (-0.24; 0.53)
IST-sTAU	-0.87	399	0.39	0.19 (-0.24; 0.61)
General quality of life[c]				
Main time effect[a]				
34	9.32	1031	<0.001	0.67 (0.53; 0.82)
51	11.44	668	<0.001	0.81 (0.67; 0.94)
104	9.87	582	<0.001	0.74 (0.59; 0.88)
34 x treatment				
GST-IST	-0.30	1009	0.77	0.05 (-0.26; 0.36)
GST-sTAU	-2.55	1044	0.01	0.41 (0.10; 0.73)
IST-sTAU	-2.01	1036	0.04	0.37 (0.01; 0.73)
51 x treatment				

**Outcome, weeks, comparison**	***t***	***df***	***P***	***d* (95%CI)**
GST-IST	1.99	648	0.047	0.33 (0.00; 0.65)
GST-sTAU	-0.71	682	0.48	0.12 (-0.21; 0.45)
IST-sTAU	-2.36	672	0.02	0.45 (0.07; 0.82)
104 x treatment				
GST-IST	0.33	553	0.74	0.06 (-0.27; 0.39)
GST-sTAU	-0.52	603	0.60	0.09 (-0.26; 0.44)
IST-sTAU	-0.75	586	0.45	0.15 (-0.24; 0.54)
Mental health quality of life[c]				
Main time effect[a]				
34	10.67	1013	<0.001	0.79 (0.64; 0.93)
51	12.08	631	<0.001	0.91 (0.77; 1.06)
104	12.06	489	<0.001	0.96 (0.81; 1.12)
34 x treatment				
GST-IST	-1.068	988	0.29	0.18 (-0.15; 0.50)
GST-sTAU	-2.781	1029	0.006	0.47 (0.14; 0.80)
IST-sTAU	-3.398	1019	<0.001	0.65 (0.27; 1.02)
51 x treatment				
GST-IST	-1.859	611	0.06	0.33 (-0.02; 0.67)
GST-sTAU	-1.018	645	0.31	0.18 (-0.17; 0.54)
IST-sTAU	-2.518	635	0.01	0.51 (0.11; 0.91)
104 x treatment				
GST-IST	-0.756	462	0.45	0.14 (-0.22; 0.50)
GST-sTAU	-0.204	509	0.84	0.04 (-0.34; 0.42)
IST-sTAU	-0.829	492	0.41	0.18 (-0.24; 0.60)
Happiness				
Main time effect[a]				
34	14.98	1161	<0.001	0.94 (0.81; 1.06)
51	15.82	1161	<0.001	1.04 (0.91; 1.17)
104	15.50	1161	<0.001	1.06 (0.93; 1.19)
34 x treatment				
GST-IST	0.51	1161	0.61	0.08 (-0.22; 0.37)
GST-sTAU	2.78	1161	0.006	0.43 (0.12; 0.73)
IST-sTAU	2.01	1161	0.04	0.35 (0.01; 0.69)
51 x treatment				
GST-IST	-0.37	1161	0.71	0.06 (-0.25; 0.37)
GST-sTAU	1.86	1161	0.06	0.30 (-0.02; 0.62)
IST-sTAU	1.98	1161	0.048	0.36 (0.00; 0.72)
104 x treatment				
GST-IST	0.12	1161	0.91	0.02 (-0.30; 0.34)
GST-sTAU	0.25	1161	0.80	0.04 (-0.29; 0.38)
IST-sTAU	0.12	1161	0.90	0.02 (-0.35; 0.40)
PD-related beliefs				
Main time effect[a]				
34	-12.30	1145	<0.001	0.69 (0.58; 0.80)
51	-13.49	1145	<0.001	0.84 (0.71; 0.96)
104	-14.56	1145	<0.001	0.97 (0.84; 1.10)

**Outcome, weeks, comparison**	***t***	***df***	***P***	***d* (95%CI)**
34 x treatment				
GST-IST	0.68	1145	0.50	0.09 (-0.17; 0.36)
GST-sTAU	-2.38	1145	0.02	0.33 (0.06; 0.60)
IST-sTAU	-2.70	1145	0.007	0.42 (0.11; 0.73)
51 x treatment				
GST-IST	1.26	1145	0.21	0.19 (-0.10; 0.48)
GST-sTAU	-1.93	1145	0.054	0.29 (-0.01; 0.59)
IST-sTAU	-2.81	1145	0.005	0.48 (0.15; 0.82)
104 x treatment				
GST-IST	0.09	1145	0.93	0.01 (-0.30; 0.32)
GST-sTAU	-1.13	1145	0.26	0.19 (-0.14; 0.51)
IST-sTAU	-1.09	1145	0.27	0.20 (-0.16; 0.56)
Self-esteem				
Main time effect[a]				
34	15.32	1159	<0.001	0.86 (0.75; 0.97)
51	14.52	1159	<0.001	0.91 (0.78; 1.03)
104	16.02	1159	<0.001	1.08 (0.95; 1.21)
34 x treatment				
GST-IST	-1.14	1159	0.25	0.15 (-0.11; 0.42)
GST-sTAU	2.55	1159	0.01	0.35 (0.08; 0.62)
IST-sTAU	3.26	1159	0.001	0.51 (0.20; 0.81)
51 x treatment				
GST-IST	-1.17	1159	0.24	0.18 (-0.12; 0.47)
GST-sTAU	2.00	1159	0.046	0.31 (0.01; 0.61)
IST-sTAU	2.80	1159	0.005	0.48 (0.14; 0.82)
104 x treatment				
GST-IST	-1.01	1159	0.31	0.16 (-0.15; 0.48)
GST-sTAU	0.65	1159	0.52	0.11 (-0.22; 0.44)
IST-sTAU	1.45	1159	0.15	0.27 (-0.10; 0.64)
Self-ideal discrepancy				
Main time effect[a]				
34	13.00	1151	<0.001	0.70 (0.59; 0.80)
51	12.68	1151	<0.001	0.75 (0.64; 0.87)
104	13.45	1151	<0.001	0.86 (0.73; 0.98)
34 x treatment				
GST-IST	-0.25	1151	0.80	0.03 (-0.22; 0.29)
GST-sTAU	1.85	1151	0.06	0.24 (-0.01; 0.50)
IST-sTAU	1.86	1151	0.06	0.28 (-0.02; 0.57)
51 x treatment				
GST-IST	-1.17	1151	0.24	0.17 (-0.11; 0.45)
GST-sTAU	0.94	1151	0.35	0.14 (-0.15; 0.43)
IST-sTAU	1.86	1151	0.06	0.31 (-0.02; 0.63)
104 x treatment				
GST-IST	-0.50	1151	0.62	0.08 (-0.22; 0.37)
GST-sTAU	0.23	1151	0.81	0.04 (-0.28; 0.35)
IST-sTAU	0.64	1151	0.52	0.11 (-0.23; 0.46)
Introversion				

**Outcome, weeks, comparison**	***t***	***df***	***P***	***d* (95%CI)**
Main time effect[a]				
34	-10.01	1147	<0.001	0.39 (0.31; 0.47)
51	-10.91	1147	<0.001	0.46 (0.38; 0.54)
104	-11.12	1147	<0.001	0.50 (0.41; 0.58)
34 x treatment				
GST-IST	-0.69	1147	0.49	0.06 (-0.12; 0.25)
GST-sTAU	-1.17	1147	0.24	0.11 (-0.08; 0.30)
IST-sTAU	-0.45	1147	0.65	0.05 (-0.16; 0.26)
51 x treatment				
GST-IST	-0.33	1147	0.74	0.03 (-0.17; 0.23)
GST-sTAU	0.05	1147	0.96	0.00 (-0.20; 0.21)
IST-sTAU	0.33	1147	0.74	0.04 (-0.19; 0.27)
104 x treatment				
GST-IST	-0.87	1147	0.39	0.09 (-0.12; 0.30)
GST-sTAU	-0.60	1147	0.55	0.07 (-0.15; 0.29)
IST-sTAU	0.20	1147	0.84	0.02 (-0.22; 0.27)
Schemas				
Main time effect[a]				
34	-13.23	1150	<0.001	0.68 (0.58; 0.79)
51	-14.24	1150	<0.001	0.84 (0.73; 0.96)
104	-14.25	1150	<0.001	0.93 (0.80; 1.05)
34 x treatment				
GST-IST	1.13	1150	0.26	0.14 (-0.10; 0.39)
GST-sTAU	-2.72	1150	0.007	0.35 (0.10; 0.59)
IST-sTAU	-3.40	1150	<0.001	0.49 (0.21; 0.77)
51 x treatment				
GST-IST	1.53	1150	0.13	0.22 (-0.06; 0.50)
GST-sTAU	-1.64	1150	0.10	0.24 (-0.05; 0.52)
IST-sTAU	-2.80	1150	0.005	0.46 (0.14; 0.78)
104 x treatment				
GST-IST	0.57	1150	0.57	0.09 (-0.21; 0.39)
GST-sTAU	-0.51	1150	0.61	0.08 (-0.24; 0.40)
IST-sTAU	-0.95	1150	0.34	0.17 (-0.18; 0.52)
Schema modes - functional				
Main time effect[a]				
34	14.19	1156	<0.001	0.83 (0.72; 0.94)
51	13.98	1156	<0.001	0.92 (0.79; 1.05)
104	15.74	1156	<0.001	1.13 (0.99; 1.27)
34 x treatment				
GST-IST	0.31	1156	0.76	0.04 (-0.23; 0.32)
GST-sTAU	4.23	1156	<0.001	0.61 (0.33; 0.89)
IST-sTAU	3.48	1156	<0.001	0.56 (0.25; 0.88)
51 x treatment				
GST-IST	-1.89	1156	0.06	0.30 (-0.01; 0.61)
GST-sTAU	2.69	1156	0.007	0.44 (0.12; 0.75)
IST-sTAU	4.05	1156	<0.001	0.74 (0.38; 1.09)

Outcome, weeks, comparison	*t*	*df*	*P*	*d* (95%CI)
104 x treatment				
GST-IST	-0.91	1156	0.36	0.15 (-0.18; 0.49)
GST-sTAU	1.56	1156	0.12	0.28 (-0.07; 0.63)
IST-sTAU	2.19	1156	0.03	0.43 (0.04; 0.82)
Schema modes - dysfunctional				
Main time effect[a]				
34	-13.98	1156	<0.001	0.73 (0.62; 0.83)
51	-14.11	1156	<0.001	0.85 (0.73; 0.96)
104	-14.24	1156	<0.001	0.95 (0.82; 1.08)
34 x treatment				
GST-IST	0.63	1156	0.53	0.08 (-0.17; 0.32)
GST-sTAU	-3.82	1156	<0.001	0.49 (0.24; 0.74)
IST-sTAU	-3.94	1156	<0.001	0.57 (0.28; 0.85)
51 x treatment				
GST-IST	1.29	1156	0.20	0.19 (-0.10; 0.47)
GST-sTAU	-2.64	1156	0.008	0.39 (0.10; 0.68)
IST-sTAU	-3.48	1156	<0.001	0.58 (0.25; 0.90)
104 x treatment				
GST-IST	0.44	1156	0.66	0.07 (-0.24; 0.38)
GST-sTAU	-1.88	1156	0.06	0.31 (0.01; 0.64)
IST-sTAU	-2.07	1156	0.04	0.38 (0.02; 0.74)
Sleep problems				
Main time effect[a]				
34	-10.87	1149	<0.001	0.49 (0.40; 0.57)
51	-11.75	1149	<0.001	0.57 (0.47; 0.66)
104	-10.71	1149	<0.001	0.55 (0.45; 0.65)
34 x treatment				
GST-IST	1.36	1149	0.18	0.15 (-0.07; 0.36)
GST-sTAU	-2.15	1149	0.03	0.24 (0.02; 0.45)
IST-sTAU	-3.09	1149	0.002	0.38 (0.14; 0.62)
51 x treatment				
GST-IST	1.13	1149	0.26	0.13 (-0.10; 0.36)
GST-sTAU	-0.61	1149	0.54	0.07 (-0.16; 0.31)
IST-sTAU	-1.52	1149	0.13	0.20 (-0.06; 0.47)
104 x treatment				
GST-IST	0.50	1149	0.61	0.06 (-0.18; 0.30)
GST-sTAU	-0.04	1149	0.10	0.01 (-0.25; 0.26)
IST-sTAU	-0.47	1149	0.64	0.07 (-0.21; 0.34)

Abbreviations: GST, Group Schema Therapy; IST, Individual Schema Therapy; sTAU, specialized treatment-as-usual.

[a] The main time effect is the weighted mean across all three conditions.

[b] Analysed with a GLMM with a log link. As the Gamma distribution is only defined for positive values and models right-skewed data, BSI scores were minimally increased so that scores are just above zero (+0.01).

[c] Analysed with a GLMM with a log link. As the Gamma distribution is only defined for positive values and models right-skewed data, left-skewed data were multiplied by -1 and minimally increased so that scores are just above zero (+0.01).

sTable 4. Estimated Means, 95%CIs, and Effect Sizes for the Primary and Secondary Outcome Variables.

	GST (n = 172)		IST (n = 96)		sTAU (n = 101)	
**Outcome, weeks**	**Estimated mean (95% CI)**	***d***	**Estimated mean (95% CI)**	***d***	**Estimated mean (95% CI)**	***d***
**Primary outcome**						
Cluster-C PD severity						
0	0.03 (-0.15; 0.21)	NA	-0.1 (-0.33; 0.12)	NA	0.01 (-0.21; 0.23)	NA
17	-0.44 (-0.6; -0.27)	0.47	-0.55 (-0.76; -0.35)	0.45	-0.37 (-0.57; -0.16)	0.38
34	-0.89 (-1.07; -0.7)	0.92	-0.99 (-1.22; -0.76)	0.89	-0.73 (-0.97; -0.5)	0.74
51	-1.07 (-1.26; -0.87)	1.10	-1.28 (-1.52; -1.04)	1.18	-0.75 (-1.00; -0.51)	0.76
104	-1.17 (-1.36; -0.97)	1.20	-1.41 (-1.65; -1.16)	1.30	-1.07 (-1.33; -0.81)	1.08
**Secondary outcomes**						
General functioning						
0	36.56 (33.99; 39.14)	NA	36.39 (33.09; 39.69)	NA	37.78 (34.55; 41.01)	NA
17	30.88 (28.5; 33.26)	0.37	30.9 (27.88; 33.92)	0.35	33.85 (30.86; 36.84)	0.25
34	25.37 (22.71; 28.03)	0.72	25.57 (22.14; 29)	0.70	30.03 (26.58; 33.49)	0.50
51	23.07 (20.32; 25.83)	0.87	21.08 (17.58; 24.58)	0.99	27.81 (24.2; 31.41)	0.64
104	23.23 (20.4; 26.07)	0.86	23.05 (19.47; 26.63)	0.86	25.4 (21.54; 29.25)	0.80
Psycho-pathology[a]						
0	1.17 (1.04; 1.32)	NA	1.14 (0.98; 1.31)	NA	1.25 (1.08; 1.44)	NA
17	0.95 (0.85; 1.06)	-0.43	0.87 (0.76; 0.99)	-0.52	1.12 (0.98; 1.28)	-0.22
34	0.77 (0.68; 0.86)	-0.84	0.67 (0.58; 0.78)	-1.03	1.00 (0.86; 1.17)	-0.43
51	0.71 (0.63; 0.81)	-0.98	0.63 (0.54; 0.73)	-1.17	0.87 (0.74; 1.02)	-0.71
104	0.69 (0.61; 0.78)	-1.05	0.65 (0.56; 0.76)	-1.09	0.79 (0.67; 0.93)	-0.90
General quality of life[a]						
0	0.62 (0.57; 0.67)	NA	0.65 (0.59; 0.70)	NA	0.61 (0.55; 0.67)	NA
17	0.71 (0.67; 0.74)	0.40	0.72 (0.68; 0.76)	0.38	0.66 (0.60; 0.70)	0.19
34	0.77 (0.74; 0.80)	0.79	0.78 (0.74; 0.82)	0.75	0.70 (0.64; 0.74)	0.38
51	0.77 (0.73; 0.80)	0.75	0.82 (0.79; 0.85)	1.07	0.74 (0.69; 0.78)	0.63
104	0.76 (0.73; 0.80)	0.74	0.79 (0.75; 0.82)	0.80	0.74 (0.69; 0.79)	0.65
Mental health quality of life[a]						
0	0.42 (0.33; 0.49)	NA	0.42 (0.31; 0.51)	NA	0.38 (0.26; 0.47)	NA
17	0.56 (0.50; 0.61)	0.44	0.58 (0.51; 0.64)	0.53	0.45 (0.36; 0.53)	0.20
34	0.66 (0.61; 0.71)	0.86	0.70 (0.64; 0.75)	1.04	0.51 (0.42; 0.60)	0.39
51	0.67 (0.61; 0.71)	0.87	0.73 (0.67; 0.78)	1.20	0.60 (0.51; 0.67)	0.69
104	0.68 (0.63; 0.72)	0.94	0.71 (0.65; 0.76)	1.07	0.65 (0.57; 0.71)	0.90

Happiness						
0	3.26 (3.09; 3.43)	NA	3.39 (3.16; 3.62)	NA	3.28 (3.05; 3.5)	NA
17	3.86 (3.71; 4.01)	0.54	3.94 (3.75; 4.14)	0.5	3.64 (3.44; 3.83)	0.32
34	4.44 (4.26; 4.62)	1.06	4.48 (4.24; 4.72)	0.99	3.98 (3.73; 4.23)	0.64
51	4.47 (4.28; 4.66)	1.10	4.66 (4.41; 4.92)	1.15	4.16 (3.89; 4.42)	0.80
104	4.45 (4.25; 4.65)	1.08	4.55 (4.3; 4.81)	1.06	4.42 (4.13; 4.7)	1.03
PD-related beliefs						
0	51.48 (48.56; 54.41)	NA	52.64 (48.97; 56.31)	NA	52.08 (48.48; 55.68)	NA
17	45.65 (42.93; 48.37)	0.38	46.08 (42.72; 49.44)	0.43	48.81 (45.46; 52.16)	0.21
34	39.99 (36.96; 43.02)	0.75	39.73 (35.93; 43.53)	0.84	45.64 (41.75; 49.54)	0.42
51	38.29 (35.18; 41.41)	0.86	36.56 (32.68; 40.45)	1.05	43.41 (39.37; 47.44)	0.56
104	35.96 (32.74; 39.17)	1.01	36.89 (32.92; 40.85)	1.02	39.42 (35.15; 43.69)	0.82
Self-esteem						
0	11.12 (10.28; 11.97)	NA	10.89 (9.79; 12)	NA	10.85 (9.77; 11.93)	NA
17	13.4 (12.63; 14.17)	0.46	13.56 (12.57; 14.55)	0.54	12.24 (11.26; 13.23)	0.28
34	15.61 (14.73; 16.49)	0.90	16.14 (15; 17.29)	1.06	13.59 (12.42; 14.76)	0.55
51	15.76 (14.85; 16.67)	0.94	16.4 (15.23; 17.58)	1.11	13.97 (12.75; 15.19)	0.63
104	16.38 (15.44; 17.33)	1.06	16.96 (15.76; 18.15)	1.22	15.57 (14.26; 16.88)	0.95
Self-ideal discrepancy						
0	-36.63 (-38.96; -34.3)	NA	-36.83 (-39.95; -33.71)	NA	-40.2 (-43.24; -37.15)	NA
17	-30.67 (-32.74; -28.59)	0.38	-30.61 (-33.38; -27.84)	0.40	-36.17 (-38.93; -33.42)	0.26
34	-24.88 (-27.33; -22.43)	0.75	-24.57 (-27.84; -21.3)	0.78	-32.27 (-35.63; -28.92)	0.50
51	-24.97 (-27.51; -22.43)	0.74	-22.54 (-25.9; -19.18)	0.91	-30.71 (-34.22; -27.2)	0.60
104	-23.33 (-25.98; -20.68)	0.85	-22.34 (-25.77; -18.91)	0.92	-27.49 (-31.25; -23.73)	0.81
Introversion						
0	44.97 (42.56; 47.38)	NA	44.76 (41.76; 47.76)	NA	46.95 (44.01; 49.89)	NA
17	41.99 (39.67; 44.31)	0.22	42.22 (39.36; 45.09)	0.19	44.75 (41.91; 47.58)	0.16
34	39.11 (36.65; 41.57)	0.44	39.77 (36.71; 42.83)	0.37	42.61 (39.52; 45.7)	0.32
51	38.7 (36.2; 41.2)	0.47	38.94 (35.83; 42.04)	0.43	40.62 (37.46; 43.78)	0.47
104	37.75 (35.21; 40.3)	0.54	38.77 (35.63; 41.91)	0.45	40.64 (37.37; 43.91)	0.47
Schemas						
0	3.21 (3.08; 3.35)	NA	3.26 (3.09; 3.43)	NA	3.32 (3.15; 3.48)	NA
17	2.95 (2.83; 3.08)	0.37	2.95 (2.79; 3.11)	0.44	3.18 (3.02; 3.34)	0.20
34	2.7 (2.56; 2.84)	0.73	2.65 (2.48; 2.83)	0.87	3.05 (2.87; 3.23)	0.39
51	2.63 (2.48; 2.77)	0.84	2.52 (2.34; 2.7)	1.06	2.9 (2.71; 3.08)	0.60
104	2.57 (2.42; 2.72)	0.92	2.55 (2.37; 2.74)	1.01	2.73 (2.54; 2.93)	0.84

Schema modes - functional						
0	2.92 (2.8; 3.03)	NA	2.91 (2.76; 3.07)	NA	2.95 (2.8; 3.1)	NA
17	3.25 (3.15; 3.36)	0.50	3.24 (3.09; 3.38)	0.48	3.08 (2.94; 3.22)	0.20
34	3.58 (3.46; 3.7)	0.99	3.55 (3.39; 3.71)	0.95	3.21 (3.04; 3.38)	0.39
51	3.55 (3.43; 3.68)	0.95	3.75 (3.58; 3.92)	1.25	3.29 (3.12; 3.47)	0.51
104	3.69 (3.56; 3.82)	1.15	3.79 (3.62; 3.96)	1.31	3.54 (3.35; 3.72)	0.88
Schema modes - dysfunctional						
0	3.01 (2.9; 3.11)	NA	3.04 (2.91; 3.17)	NA	2.98 (2.85; 3.11)	NA
17	2.79 (2.69; 2.89)	0.42	2.8 (2.68; 2.93)	0.46	2.89 (2.77; 3.02)	0.17
34	2.58 (2.47; 2.69)	0.83	2.57 (2.44; 2.71)	0.90	2.81 (2.67; 2.95)	0.34
51	2.55 (2.44; 2.66)	0.89	2.49 (2.35; 2.62)	1.08	2.72 (2.58; 2.87)	0.50
104	2.49 (2.38; 2.6)	1.01	2.49 (2.35; 2.62)	1.08	2.63 (2.48; 2.77)	0.70
Sleep problems						
0	86.55 (83.63; 89.47)	NA	88.39 (84.71; 92.07)	NA	89.33 (85.72; 92.94)	NA
17	82.29 (79.52; 85.05)	0.26	82.89 (79.43; 86.35)	0.33	87.07 (83.64; 90.5)	0.14
34	78.15 (75.15; 81.15)	0.50	77.56 (73.78; 81.34)	0.65	84.87 (81.04; 88.7)	0.27
51	77.4 (74.33; 80.46)	0.55	77.06 (73.2; 80.91)	0.68	81.39 (77.43; 85.34)	0.48
104	77.7 (74.56; 80.84)	0.53	78.52 (74.61; 82.43)	0.59	80.56 (76.43; 84.69)	0.53

Abbreviations: GST, Group Schema Therapy; IST, Individual Schema Therapy; sTAU, specialized treatment-as-usual.

[a] Estimated means reflect scores in the original scale, Cohen's *d* effect sizes are based on the estimated means from the GLMM in the transformed scale.

Supplement 7. Analyses with Cluster-C Diagnosis

A sensitivity analysis was conducted to check whether the same results would be found for the primary outcome variable when diagnosis was added to the model. An additional goal was to see whether treatment effects were smaller for OCPD compared to APD, as was found in the GST pilot study (d = 0.59 and 1.33 respectively[41]). Due to the small sample size of DPD (N=10) and the specific interest in the comparison between APD and OCPD, only a centered dummy (APD vs. OCPD), and the interactions of this dummy with treatment, time, and treatment by time were added to the model of the severity index.

In this model, the decrease in severity scores at 51-wks compared to sTAU was again significantly larger for GST (Cohen d = 0.30, 95% CI [0.02; 0.58], p = 0.04) as well as IST (Cohen d = 0.37, 95% CI [0.06; 0.69], p = 0.02).

There was no significant overall difference between APD and OCPD in cluster-C PD severity reduction over time. This also held within the treatments, where the treatment effects of GST were large for both APD and OCPD at 51-wks (APD: Cohen d = 1.09, 95% CI [0.90; 1.29], OCPD: Cohen d = 1.09, 95% CI [0.75; 1.42]) and 2-yr follow-up (APD: Cohen d = 1.23, 95% CI [1.02; 1.44], OCPD: Cohen d = 1.03, 95% CI [0.68; 1.39]). For IST, treatment effects were also large for both APD and OCPD at 51-wks (APD: Cohen d = 1.15, 95% CI [0.90; 1.40], OCPD: Cohen d = 1.22, 95% CI [0.74; 1.71]) and 2-yr follow-up (APD: Cohen d = 1.24, 95% CI [0.99; 1.50], OCPD: Cohen d = 1.54, 95% CI [1.03; 2.05]). For sTAU, treatment effects at 51-wks were medium for APD (Cohen d = 0.67, 95% CI [0.42; 0.92]) and large for OCPD (Cohen d = 1.24, 95% CI [0.69; 1.79]) and large for both APD and OCPD at 2-yr follow-up (APD: Cohen d = 0.98, 95% CI [0.71; 1.25], OCPD: Cohen d = 1.54, 95% CI [0.94; 2.13]).

Within APD however, PD severity reduction was larger in GST than in sTAU at 34 and 51-wks. Similarly, the effects were significantly larger in IST than in sTAU for APD at 51-wks. Within OCPD, PD severity reduction did not significantly differ between treatments. There was only one significant three-way interaction: at 34-wks, the difference in improvements between APD and OCPD within sTAU was larger than the difference between APD and OCPD within GST. Interaction effects with diagnosis can be found in sTable 5 and estimated means over time for each diagnosis within treatment as well as for each treatment within diagnosis are presented in sFigure 1 and sFigure 2 respectively.

Discussion

The short-term superiority of GST and IST over sTAU found in the main analysis remained when the analysis was controlled for primary diagnosis. The effect size for APD was similar to that in the GST pilot study, but the effect size for OCPD was larger in the current study. Although we found evidence for short-term superiority of GST and IST over sTAU for APD, we did not find any differences between treatments for OCPD. From the significant three-way interaction it would appear that sTAU might be differentially effective for OCPD than APD, but the non-significant difference between APD and OCPD within sTAU does not support this notion. The small number of OCPD patients (especially within IST and sTAU) makes it difficult to draw definitive conclusions on the relative effectiveness of treatments for OCPD, and on differences between APD and OCPD within treatments. Hence, future research should further explore these differences in adequately powered analyses.

sTable 5. Sensitivity analysis with cluster c diagnosis added to the Severity Index LMM

	t	*df*	*P*	*d (95% CI)*
**Overall effect time x diagnosis**				
34 wks: OCPD vs APD	0.12	1120	0.91	0.02 (-0.24; 0.27)
51 wks: OCPD vs APD	-1.13	1120	0.26	0.16 (-0.12; 0.44)
104 wks: OCPD vs APD	-0.84	1120	0.40	0.13 (-0.17; 0.43)
**Time x diagnosis within treatment**				
**GST**				
34 wks: OCPD vs APD	1.38	1120	0.17	0.25 (-0.10; 0.61)
51 wks: OCPD vs APD	0.02	1120	0.98	0.00 (-0.38; 0.39)
104 wks: OCPD vs APD	0.96	1120	0.34	0.20 (-0.21; 0.61)
**IST**				
34 wks: OCPD vs APD	0.03	1120	0.98	0.01 (-0.49; 0.51)
51 wks: OCPD vs APD	-0.26	1120	0.80	0.07 (-0.47; 0.61)
104 wks: OCPD vs APD	-1.02	1120	0.31	0.30 (-0.27; 0.87)
**sTAU**				
34 wks: OCPD vs APD	-1.50	1120	0.13	0.42 (-0.13; 0.96)
51 wks: OCPD vs APD	-1.86	1120	0.06	0.57 (-0.03; 1.17)
104 wks: OCPD vs APD	-1.67	1120	0.09	0.56 (-0.10; 1.21)
**Time x treatment within diagnosis**				
**APD**				
34 wks x treatment				
GST vs IST	-0.69	1120	0.49	0.10 (-0.19; 0.39)
GST vs sTAU	-2.12	1120	0.03	0.31 (0.02; 0.59)
IST vs sTAU	-1.27	1120	0.20	0.21 (-0.11; 0.53)
51 wks x treatment				
GST vs IST	0.35	1120	0.72	0.06 (-0.26; 0.37)
GST vs sTAU	-2.64	1120	0.008	0.42 (0.11; 0.74)
IST vs sTAU	-2.69	1120	0.007	0.48 (0.13; 0.83)
104 wks x treatment				
GST vs IST	0.06	1120	0.95	0.01 (-0.32; 0.34)
GST vs sTAU	-1.45	1120	0.15	0.25 (-0.09; 0.59)
IST vs sTAU	-1.37	1120	0.17	0.26 (-0.11; 0.64)
**OCPD**				
34 wks x treatment				
GST vs IST	0.52	1120	0.61	0.14 (- 0.40; 0.68)
GST vs sTAU	1.21	1120	0.23	0.36 (-0.22; 0.95)
IST vs sTAU	0.64	1120	0.52	0.22 (-0.45; 0.88)
51 wks x treatment				
GST vs IST	0.44	1120	0.66	0.13 (-0.46; 0.72)
GST vs sTAU	0.46	1120	0.64	0.15 (-0.49; 0.79)
IST vs sTAU	0.05	1120	0.96	0.02 (-0.71; 0.75)
104 wks x treatment				
GST vs IST	1.61	1120	0.11	0.51 (- 0.11; 1.12)
GST vs sTAU	1.43	1120	0.15	0.50 (-0.19; 1.19)
IST vs sTAU	0.01	1120	0.99	0.00 (-0.78; 0.78)
**Three-way interaction**				
**GST vs IST**				
34 wks: OCPD vs APD	0.78	1120	0.44	0.37 (-0.57; 1.32)
51 wks: OCPD vs APD	0.22	1120	0.82	0.08 (-0.60; 0.76)
104 wks: OCPD vs APD	1.39	1120	0.16	0.25 (-0.10; 0.60)

	t	*df*	*P*	*d (95% CI)*
**GST vs sTAU**				
34 wks: OCPD vs APD	2.01	1120	0.04	1.03 (0.03; 2.03)
51 wks: OCPD vs APD	1.58	1120	0.12	0.59 (-0.14; 1.31)
104 wks: OCPD vs APD	1.93	1120	0.054	0.38 (-0.01; 0.76)
**IST vs sTAU**				
34 wks: OCPD vs APD	1.13	1120	0.26	0.65 (-0.48; 1.79)
51 wks: OCPD vs APD	1.21	1120	0.23	0.51 (-0.32; 1.34)
104 wks: OCPD vs APD	0.59	1120	0.56	0.13 (-0.30; 0.56)

Abbreviations: GST, Group Schema Therapy; IST, Individual Schema Therapy; sTAU, specialized treatment-as-usual; APD, Avoidant personality disorder; OCPD, Obsessive-compulsive personality disorder.

sFigure1. Change in Severity Index Scores per Diagnosis within Treatment

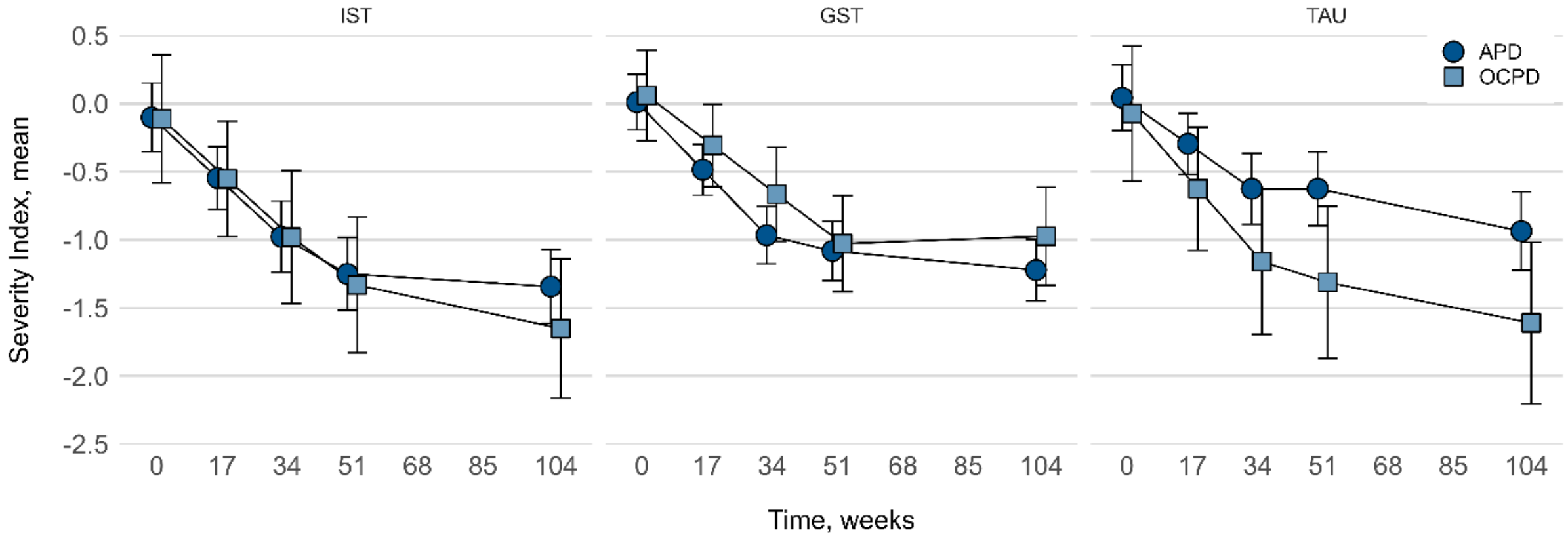

Abbreviations: GST, Group Schema Therapy; IST, Individual Schema Therapy; sTAU, specialized treatment-as-usual; APD, Avoidant personality disorder; OCPD, Obsessive-compulsive personality disorder.

sFigure 2. Change in Severity Index Scores per Treatment within Diagnosis

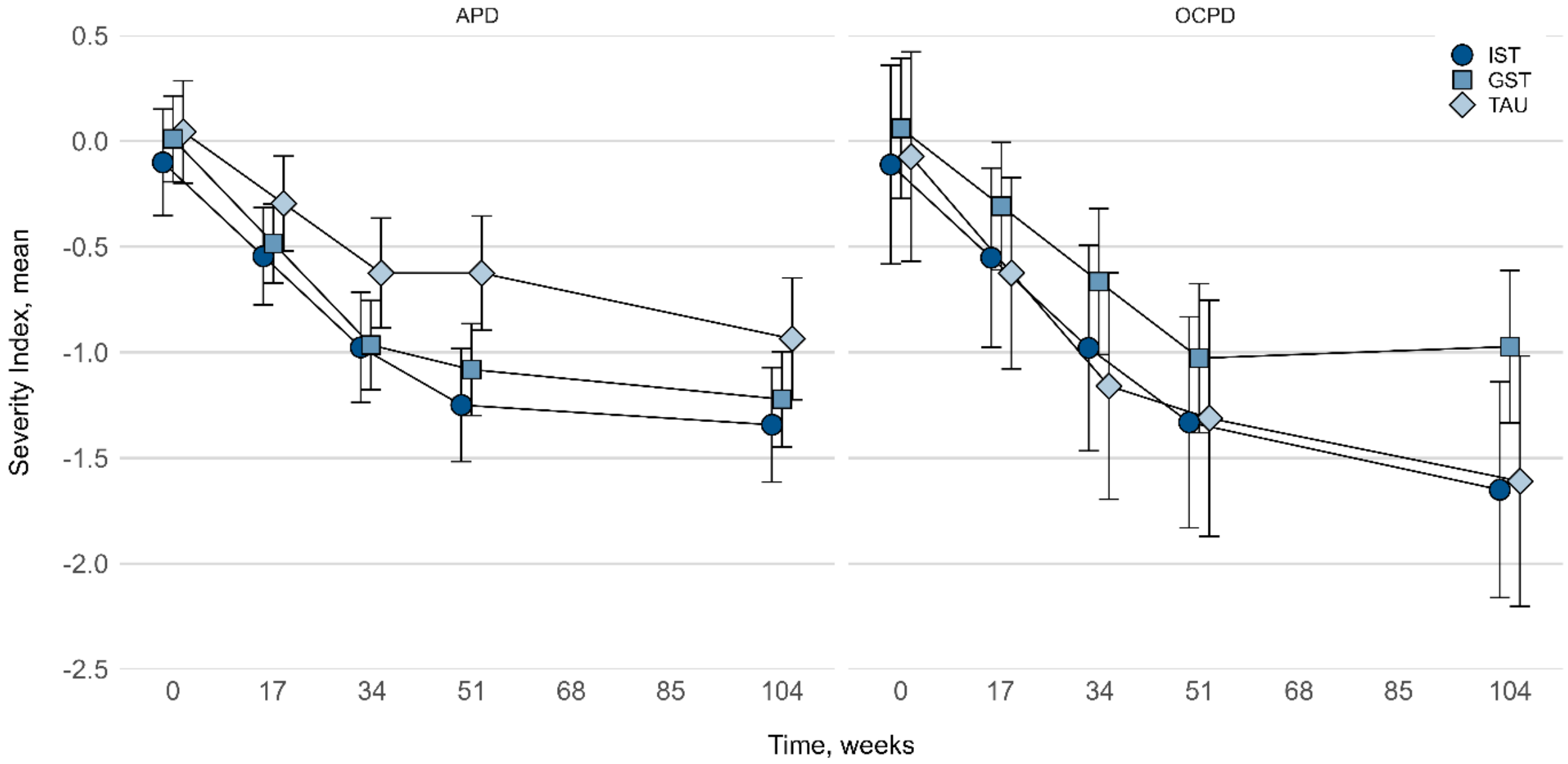

Abbreviations: GST, Group Schema Therapy; IST, Individual Schema Therapy; sTAU, specialized treatment-as-usual; APD, Avoidant personality disorder; OCPD, Obsessive-compulsive personality disorder.

Supplement 8. Sensitivity Analysis with Demographic Variables

Due to randomisation, significant differences in demographic characteristics between treatments were not expected and therefore not tested. However, sTAU did seem to deviate from the GST and IST groups somewhat, in particular regarding Living situation (with partner), Ethnic background (Dutch) and Employment status (working). To check whether results for the primary outcome variable weren't influenced by group differences on demographic characteristics, these three variables were centered and added to the model of the severity index. Similar results were found: again the decrease in severity scores at post-booster was significantly larger for IST compared to sTAU (Cohen d = 0.41, 95% CI [0.10; 0.72], p = 0.010) as well as larger for GST compared to sTAU (Cohen d = 0.33, 95% CI [0.05; 0.61], p = 0.02).

Supplement 9. Sensitivity Analysis with Missingness at Follow-up

To check whether results at follow-up could have been influenced by missing data (sTAU: 43.56%, GST: 29.07%, IST: 20.83%), a binary variable indicating whether a participant had or had not completed follow-up was added to the severity index model. Regarding the interaction of time and treatment, again the decrease in severity scores was significantly larger at post-booster for IST compared to sTAU (Cohen d = 0.38, 95% CI [0.05; 0.71], p = 0.02), but the difference between GST and sTAU was no longer significant (Cohen d = 0.28, 95% CI [-0.57; 0.00], p = 0.053).

Within treatment, no significant difference in symptom trajectories of patients with and without a follow-up assessment was found at post-treatment for sTAU (Cohen d = 0.12, 95% CI [-0.32; 0.56], p = 0.59), GST (Cohen d = 0.28, 95% CI [-0.09; 0.65], p = 0.13) or IST (Cohen d = 0.00, 95% CI [-0.61; 0.62], p >.99), nor at post-booster (sTAU: Cohen d = 0.11, 95% CI [-0.43; 0.66], p = 0.69, GST: Cohen d = 0.11, 95% CI [-0.37; 0.58], p = 0.65, IST: Cohen d = 0.04, 95% CI [-0.77; 0.86], p = 0.92).

No significant three-way interactions were found for the difference in baseline – post-treatment slopes of those with and without a follow-up assessment within sTAU compared to within GST (b= -0.62, Cohen d= 0.40, 95% CI [-0.17; 0.97], p = 0.17), within sTAU compared to within IST (Cohen d = 0.12 , 95% CI [-0.63; 0.88], p = 0.75), and within IST compared to within GST (Cohen d = 0.28, 95% CI [-0.46; 0.10], p = 0.44). This was also the case for the slopes between baseline - post-booster (sTAU - IST: Cohen d = 0.07, 95% CI [-0.92; 10.50], p = 0.90, sTAU - GST: Cohen d = 0.22, 95% CI [-0.50; 0.94], p = 0.55, IST – GST: Cohen d = 0.15, 95% CI [-0.79; 1.10], p = 0.75).

Supplement 10. Sensitivity Analyses with Treatment Sessions

At follow-up, 58.93% of patients in sTAU, 25.20% in GST, and 19.18% in IST indicated having received treatment (within or outside the study) after the post-booster assessment (average amount of sessions between post-booster and follow-up for those that received treatment: sTAU: M = 27.5, SD = 33.9, GST: M = 22.9, SD = 48.9, IST: M = 12.4, SD = 14.1). Regarding the type of follow-up care patients received after post-booster, reported answers of participants were sometimes ambiguous (“talked to a psychologist”) and varied widely. As we deemed a summary of these answers insufficiently clear and informative, we decided not to report it. To check whether results at follow-up could have been influenced by (the number of) sessions received after the post-booster assessment, two sensitivity analyses were performed.

When adding the number of treatment sessions received after post-booster to the model of the severity index, similar results were found regarding the interaction of time and treatment: again the decrease in severity scores between baseline and post-booster was significantly smaller for sTAU compared to IST (Cohen d = 0.29, 95% CI [0.05; 0.53], p = 0.02) as well as GST (Cohen d = 0.27, 95% CI [0.06; 0.49], p = 0.01). No significant additional effect of treatment sessions on symptom decrease at follow-up was found for sTAU (Cohen d = 0.003, 95% CI [-0.003; 0.009], p = 0.30), or IST (Cohen d = 0.014, 95% CI [-0.006; 0.035], p = 0.18). For GST, there was a significant additional effect (Cohen d = 0.005, 95% CI [0.001; 0.010], p = 0.03), where more additional therapy sessions after post-booster resulted in less symptom decrease. There was no significant three-way interaction for the difference in the effect of treatment sessions received after post-booster between sTAU and GST (Cohen d = 0.001, 95% CI [-0.004; 0.006], p = 0.58), sTAU and IST (Cohen d = 0.007, 95% CI [-0.007; 0.02], p = 0.31), or IST and GST (Cohen d = 0.009, 95% CI [-0.012; 0.030], p = 0.41).

When analyzed on post-booster and follow-up data only, no significant difference in symptom trajectories of patients that did or did not receive treatment after post-booster (binary variable) was found within sTAU (Cohen d = 0.26, 95% CI[-0.15; 0.66], p = 0.21) nor within GST (Cohen d = 0.13, 95% CI [-0.18; 0.44], p = 0.40) or IST (Cohen d = 0.20, 95% CI [-0.24; 0.64], p = 0.38). No significant three-way interactions were found for the difference in symptom trajectories of those with and without treatment after post-booster within sTAU compared to within GST (Cohen d = 0.12, 95% CI [-0.38; 0.63], p = 0.63), sTAU and IST (Cohen d = 0.06, 95% CI [-0.54; 0.66], p = 0.85), or IST and GST (Cohen d = 0.06, 95% CI [-0.50; 0.60], p = 0.47).

sTable 6. Number of allocated treatment sessions in each treatment arm per assessment

	GST		IST		sTAU	
	N	Sessions, mean (SD)	N	Sessions, mean (SD)	N	Sessions, mean (SD)
17 wks	155	17.15 (3.43)	88	30.27 (2.89)	84	14.68 (6.17)
34 wks	147	31.17 (4.64)	83	44.11 (3.55)	72	29.47 (15.21)
51 wks	136	35.2 (4.46)	79	48.7 (4.70)	70	41.8 (27.70)
104 wks	123	35.6 (3.66)	75	48.9 (5.51)	58	54.4 (47.82)
**Allocated treatment + treatment outside study between 51 – 104 weeks**						
104 wks	123	41.4 (27.43)	73	51.0 (10.14)	56	60.3 (49.67)

Abbreviations: GST, Group Schema Therapy; IST, Individual Schema Therapy; sTAU, specialized treatment-as-usual.

sTable 7. Sensitivity Analysis Results Cluster-C PD Severity without Outliers and Influential Cases

**Comparison**	***t***	***df***	***P***	***d (95%CI)***
Main time effect				
34 wks	-16.94	1079	<0.001	0.88 (0.78; 0.98)
51 wks	-18.93	1079	<0.001	1.07 (0.96; 1.18)
104 wks	-20.41	1079	<0.001	1.22 (1.10; 1.34)
34 wks x treatment				
GST vs IST	-0.38	1079	0.71	0.05 (-0.20; 0.29)
GST vs sTAU	-0.95	1079	0.34	0.12 (-0.13; 0.38)
IST vs sTAU	-0.51	1079	0.61	0.08 (-0.21; 0.37)
51 wks x treatment				
GST vs IST	0.59	1079	0.56	0.08 (-0.19; 0.35)
GST vs sTAU	-1.88	1079	0.06	0.27 (-0.01; 0.54)
IST vs sTAU	-2.15	1079	0.03	0.35 (0.03; 0.66)
104 wks x treatment				
GST vs IST	0.44	1079	0.66	0.06 (-0.21; 0.34)
GST vs sTAU	-0.52	1079	0.60	0.08 (-0.22; 0.38)
IST vs sTAU	-0.83	1079	0.41	0.14 (-0.20; 0.48)

Abbreviations: GST, Group Schema Therapy; IST, Individual Schema Therapy; sTAU, specialized treatment-as-usual. Seven participants identified as influential cases and one single assessment identified as an outlier were removed before rerunning the analysis.

sTable 8. Sensitivity Analysis Results Happiness without Outliers and Influential Cases

Comparison	*t*	*df*	*P*	*d (95%CI)*
Main time effect				
34 wks	15.31	1135	<0.001	0.95 (0.83; 1.07)
51 wks	16.41	1135	<0.001	1.07 (0.94; 1.20)
104 wks	15.79	1135	<0.001	1.07 (0.94; 1.21)
34 wks x treatment				
GST vs IST	0.52	1135	0.61	0.08 (-0.21; 0.37)
GST vs sTAU	2.83	1135	0.005	0.44 (0.14; 0.75)
IST vs sTAU	2.08	1135	0.04	0.36 (0.02; 0.71)
51 wks x treatment				
GST vs IST	-0.23	1135	0.82	0.04 (-0.27; 0.34)
GST vs sTAU	1.91	1135	0.06	0.31 (-0.01; 0.63)
IST vs sTAU	1.90	1135	0.06	0.35 (-0.01; 0.71)
104 wks x treatment				
GST vs IST	0.18	1135	0.86	0.03 (-0.28; 0.34)
GST vs sTAU	0.44	1135	0.66	0.08 (-0.27; 0.42)
IST vs sTAU	0.25	1135	0.80	0.05 (-0.33; 0.42)

Abbreviations: GST, Group Schema Therapy; IST, Individual Schema Therapy; sTAU, specialized treatment-as-usual. Twenty-two participants identified as influential cases and two single assessments identified as outliers were removed before rerunning the analysis.

sFigure 3. Change in secondary outcome variables

A. General functioning: WHODAS-II

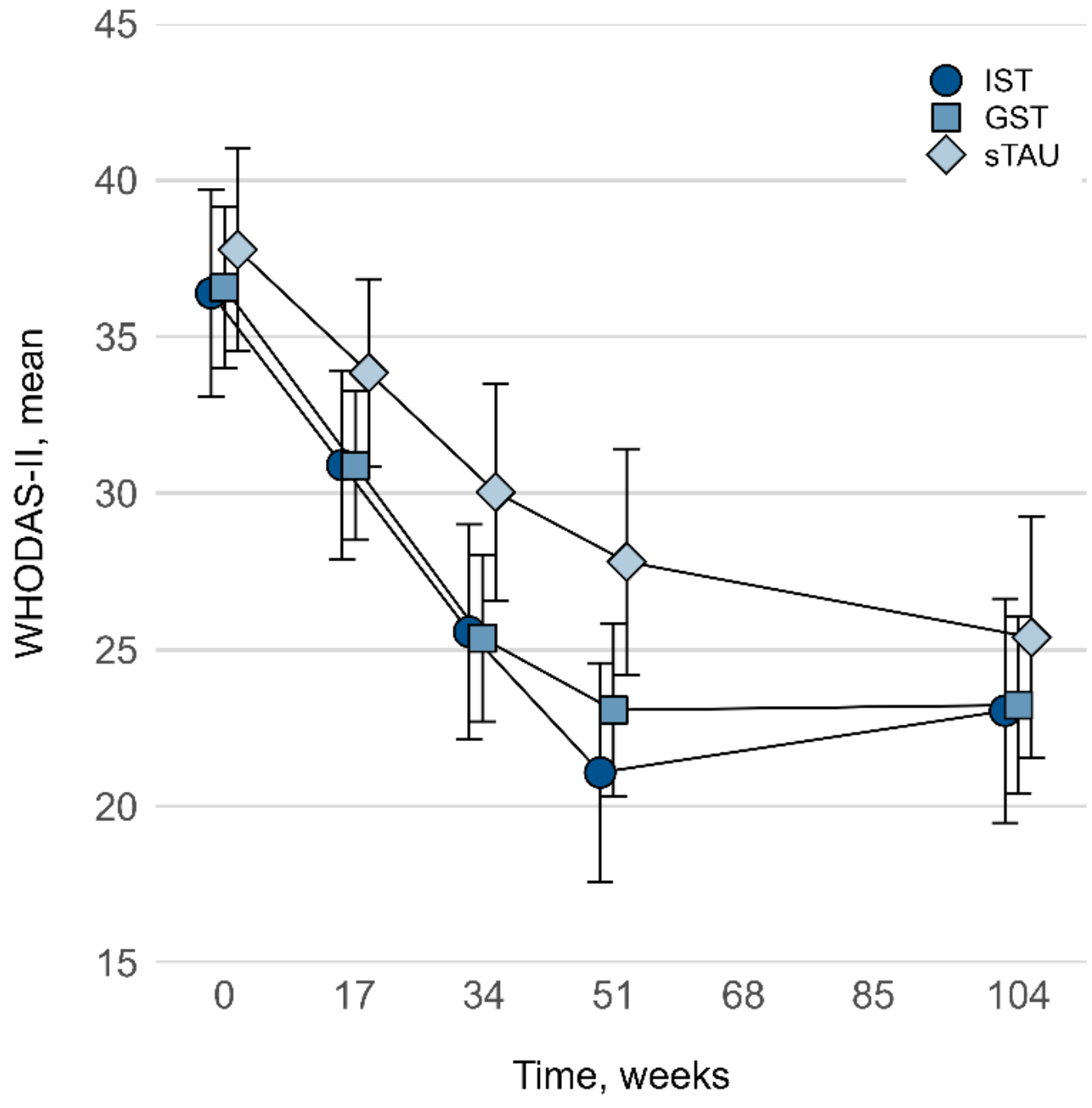

B. Psychopathology: BSI

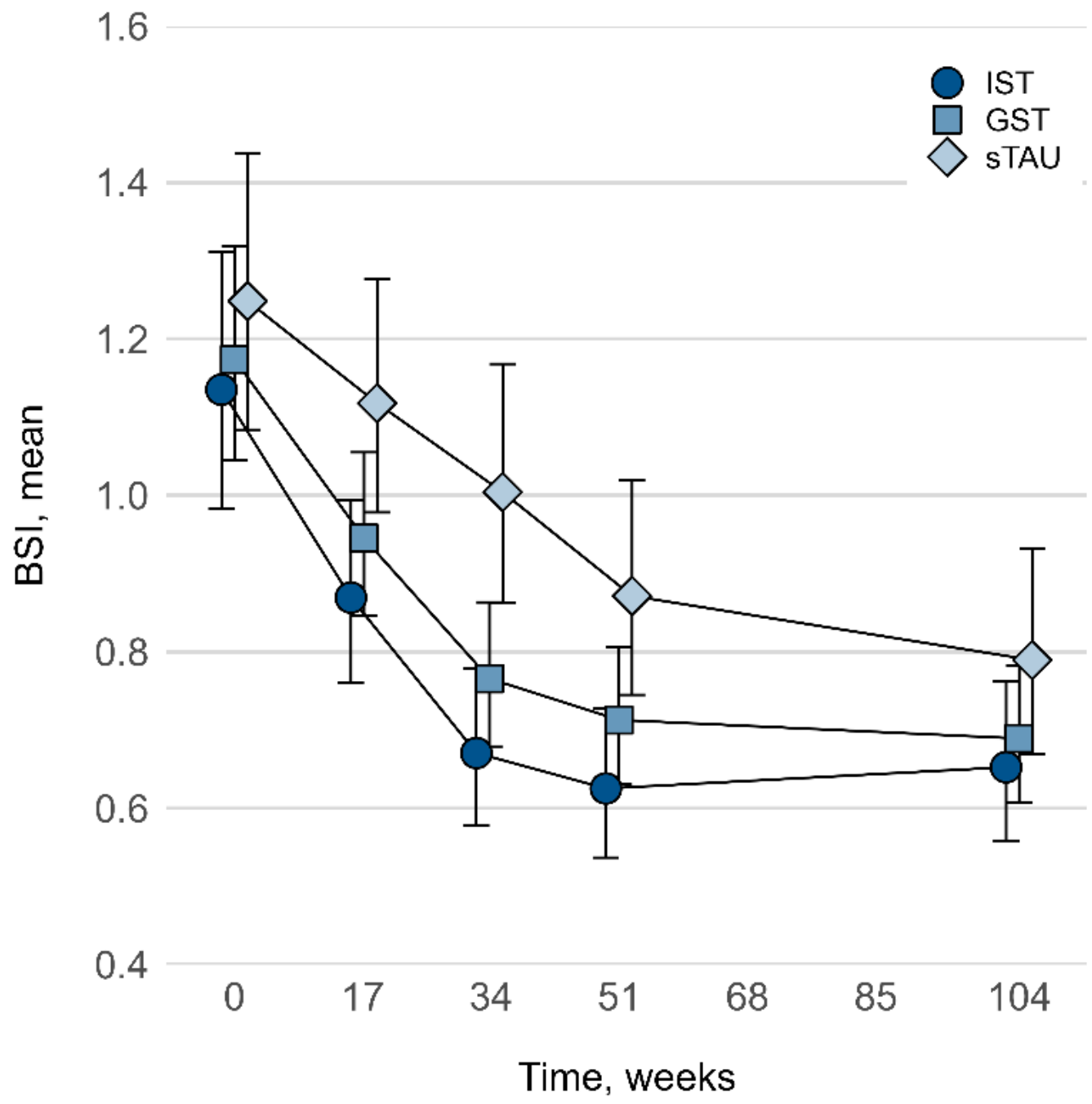

C. General quality of life: EQ-5D-5L

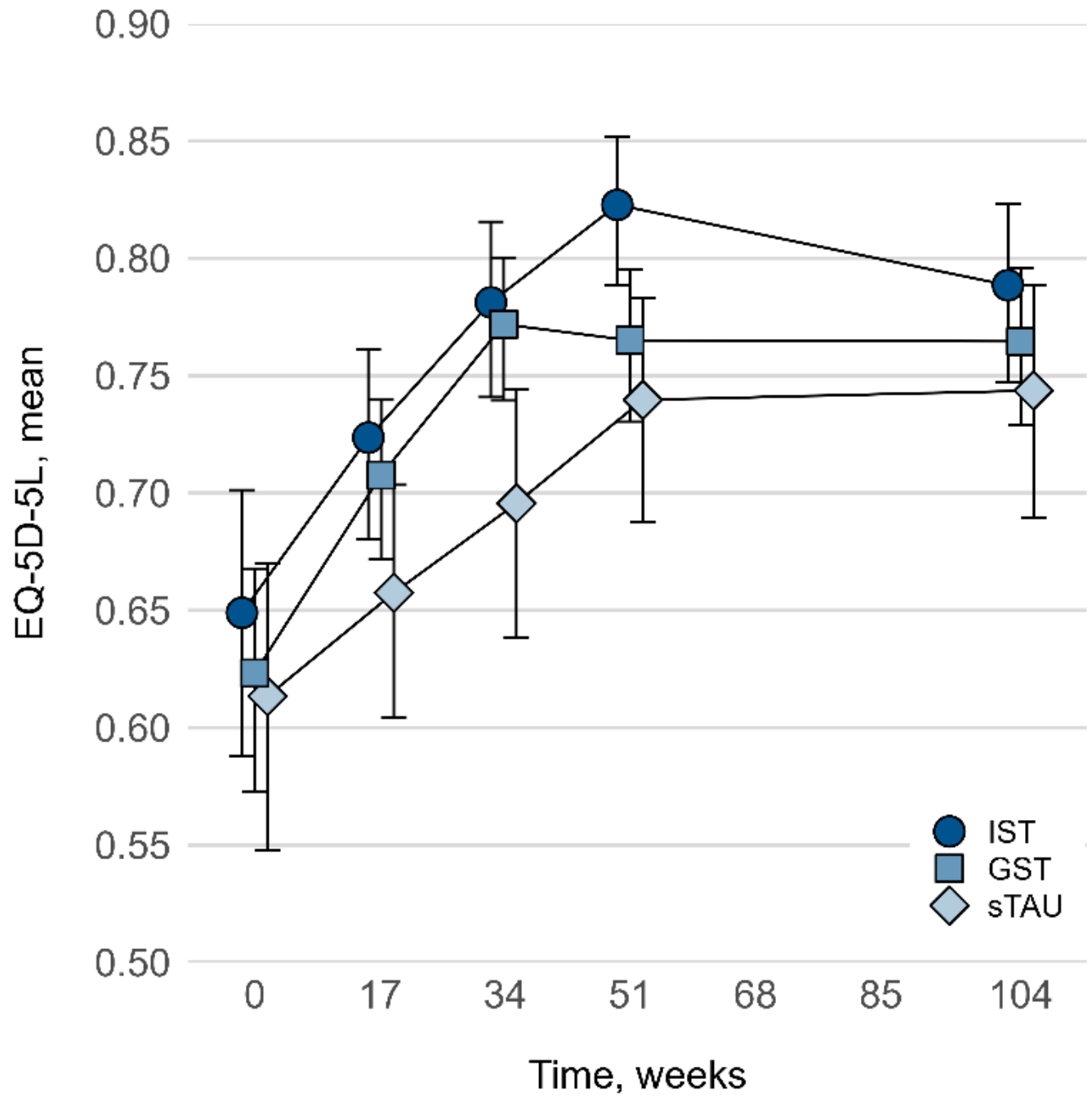

D. Mental health quality of life: MHQoL-7D

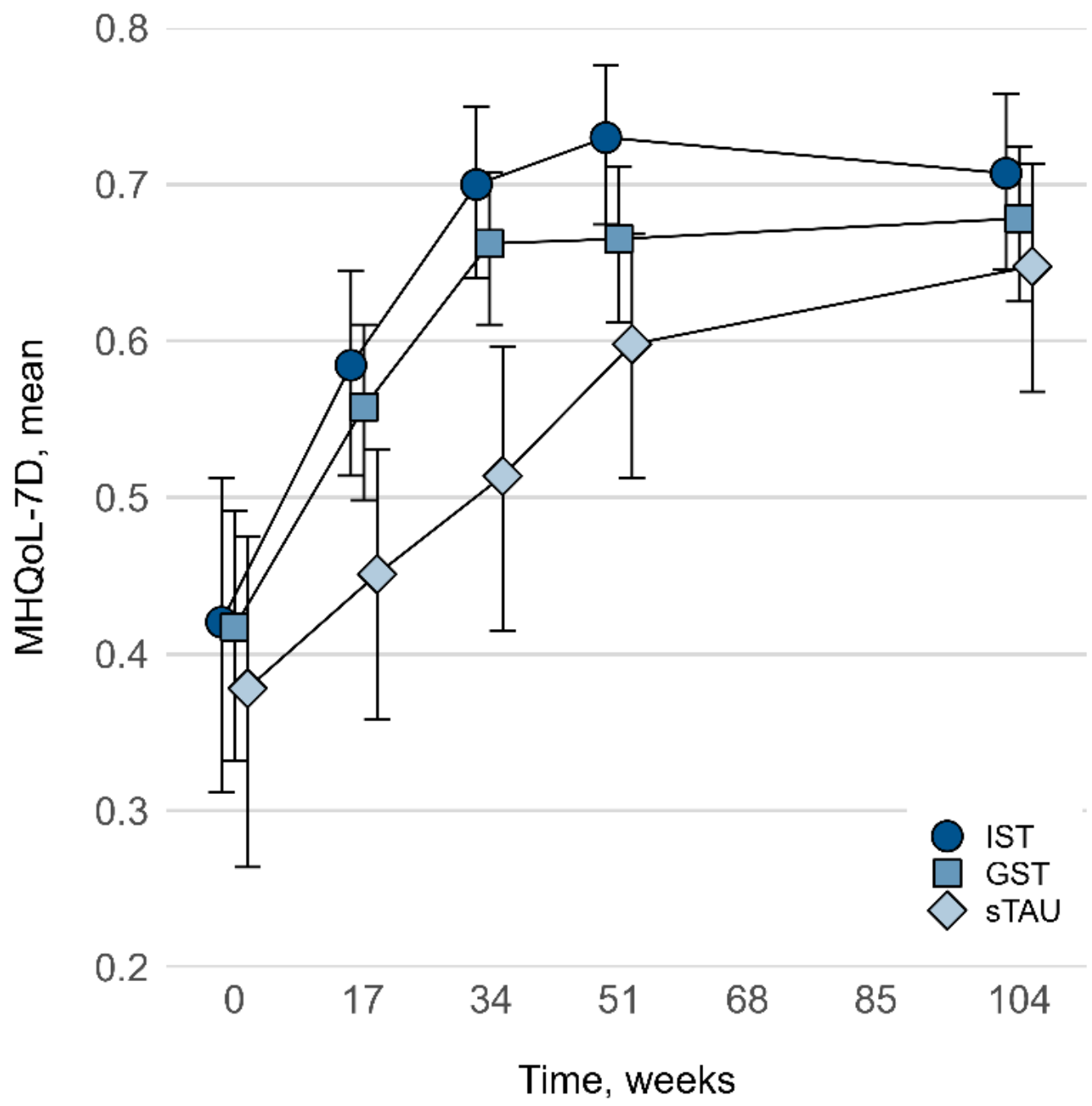

E. Happiness

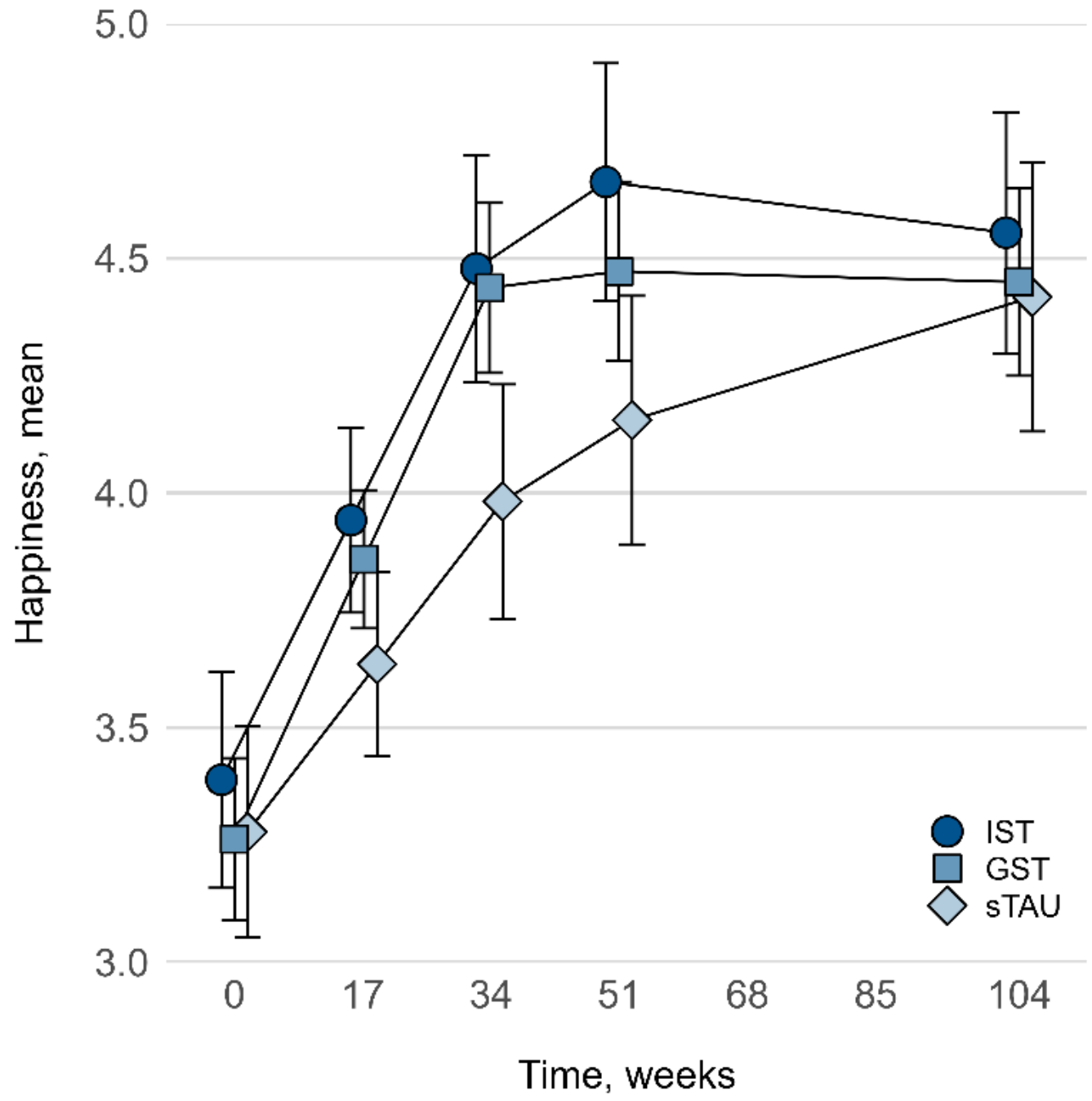

F. PD-related beliefs: PDBQ

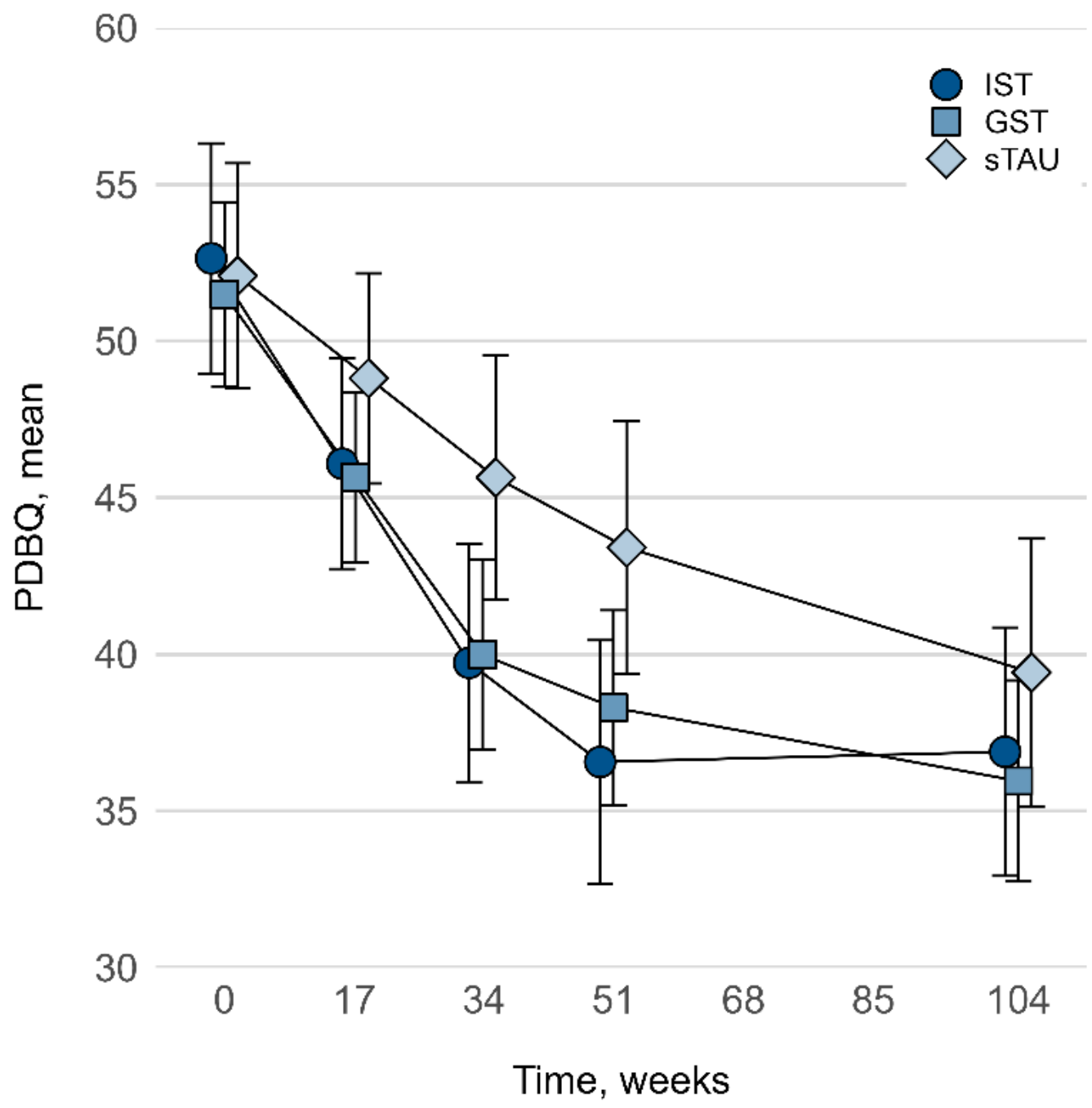

G. Self-esteem: RSES

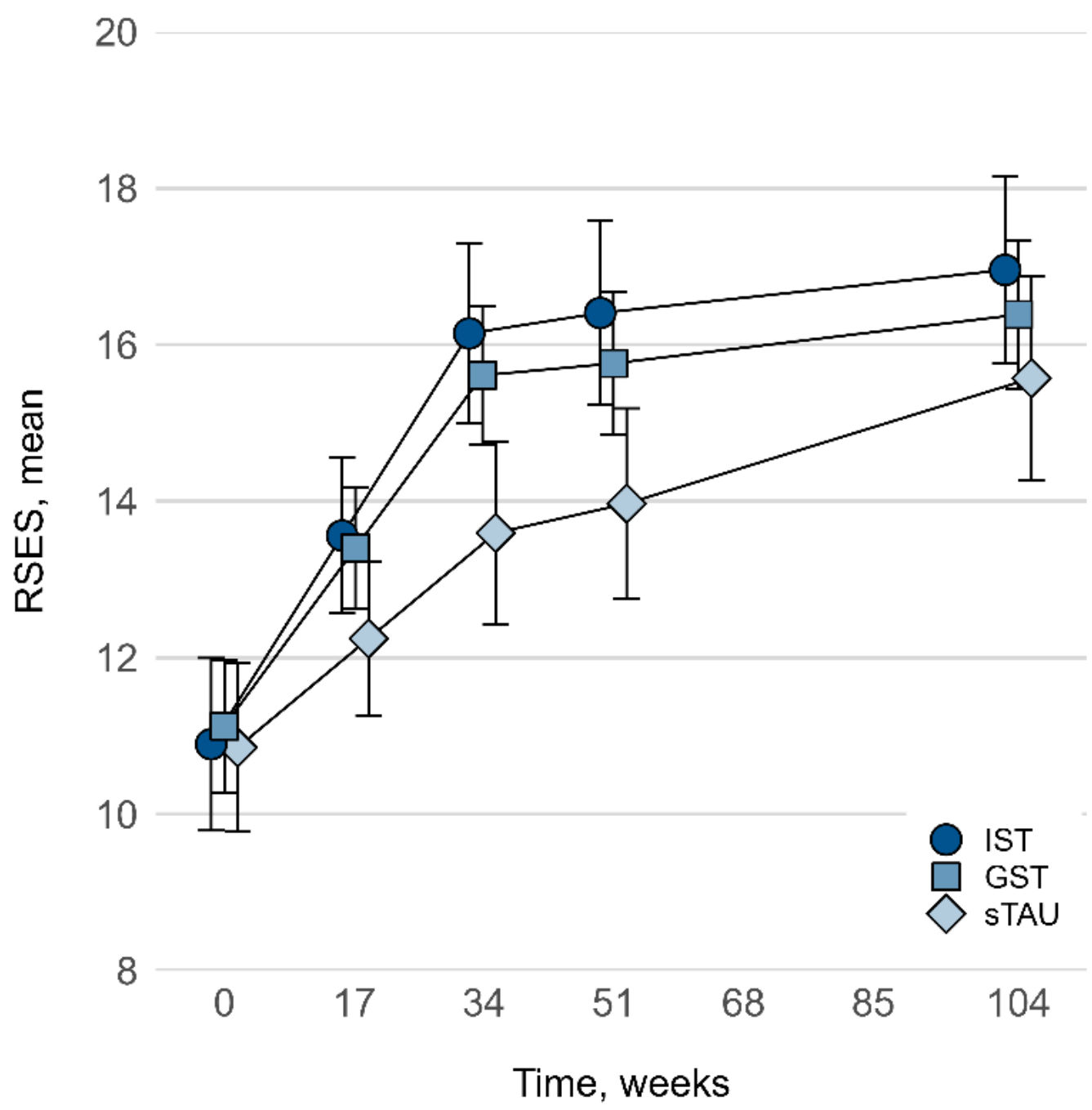

H. Self-ideal discrepancy: MSGO

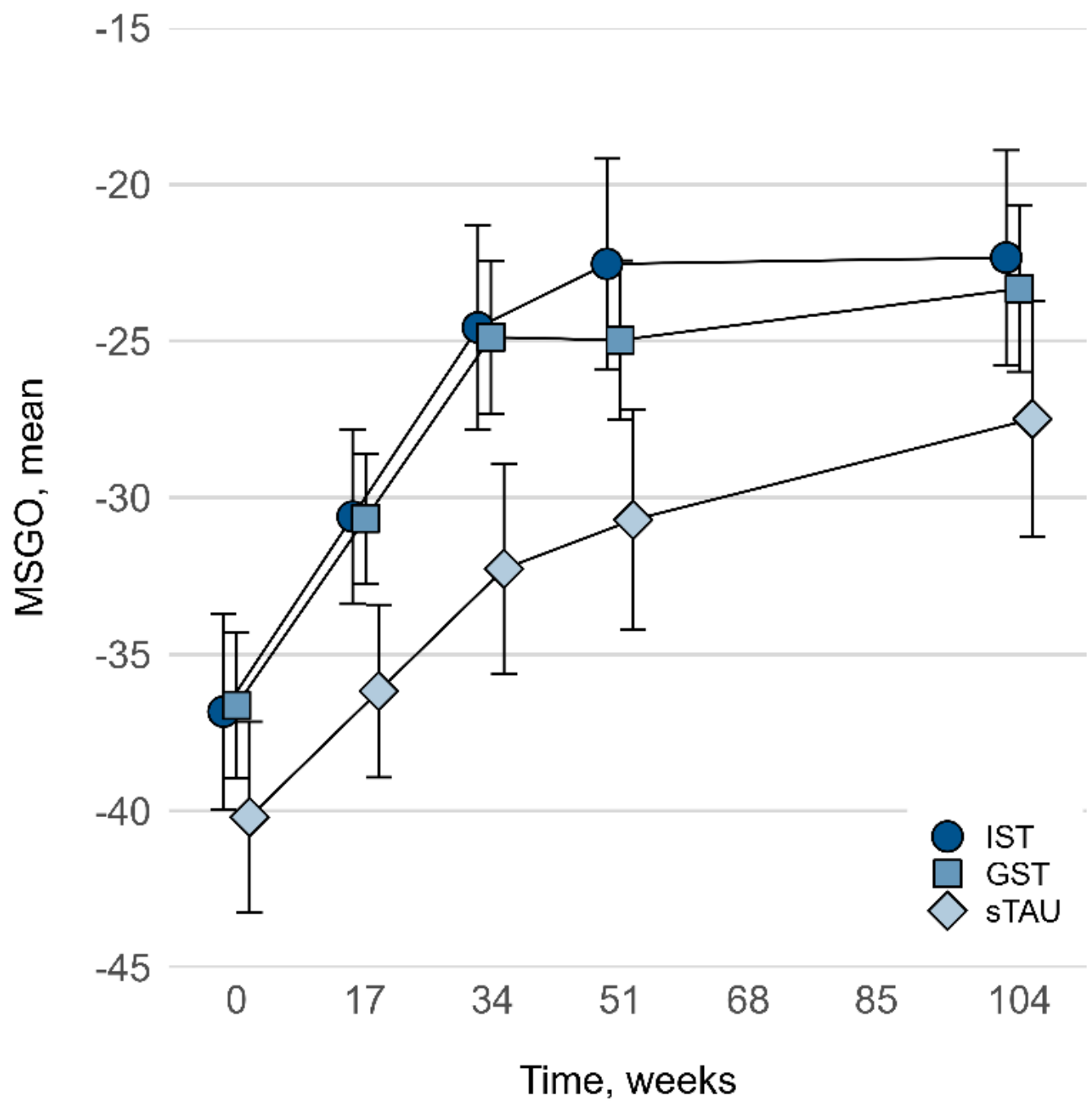

I. Introversion Inventory

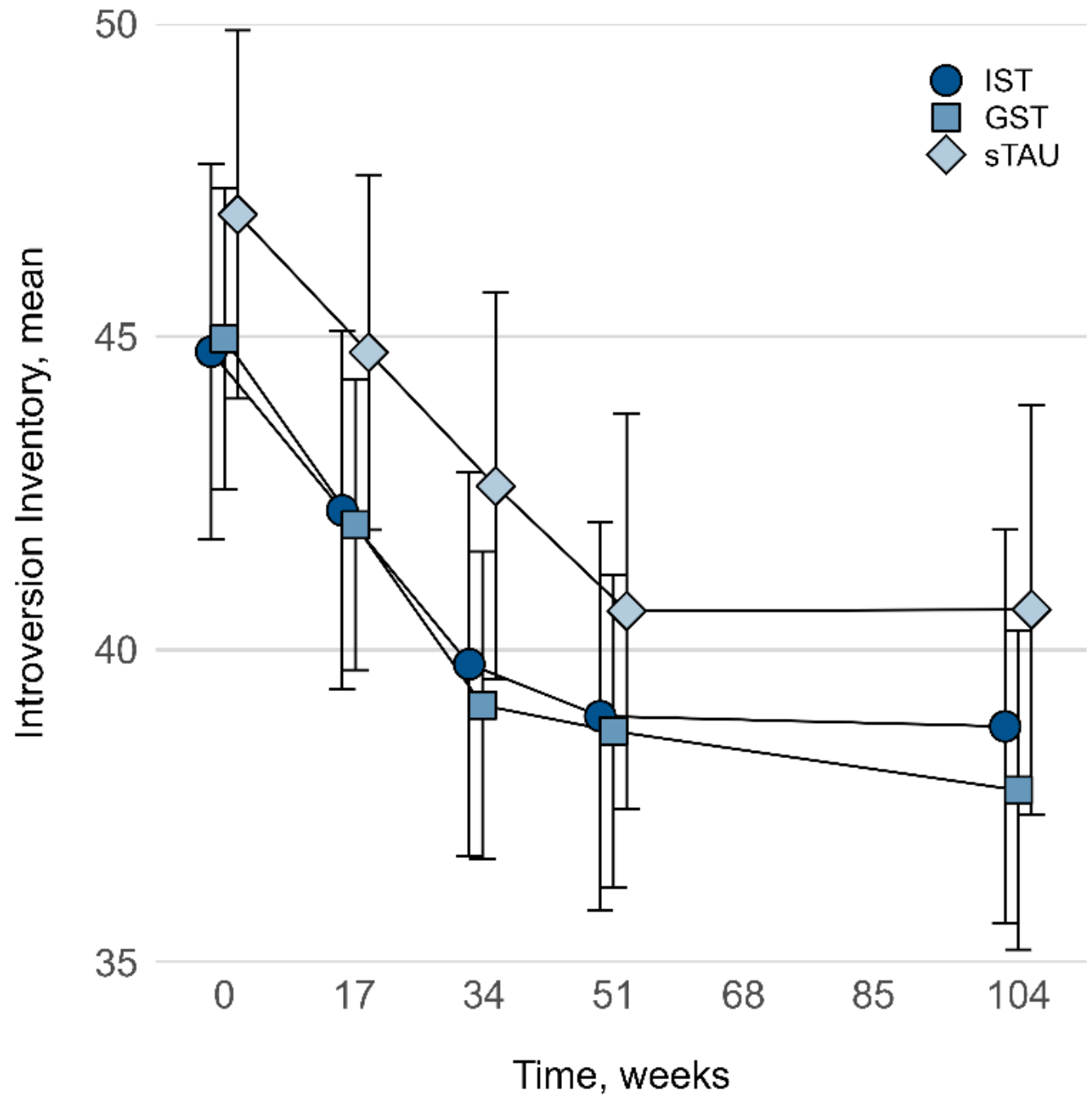

J. Schemas: YSQ-R

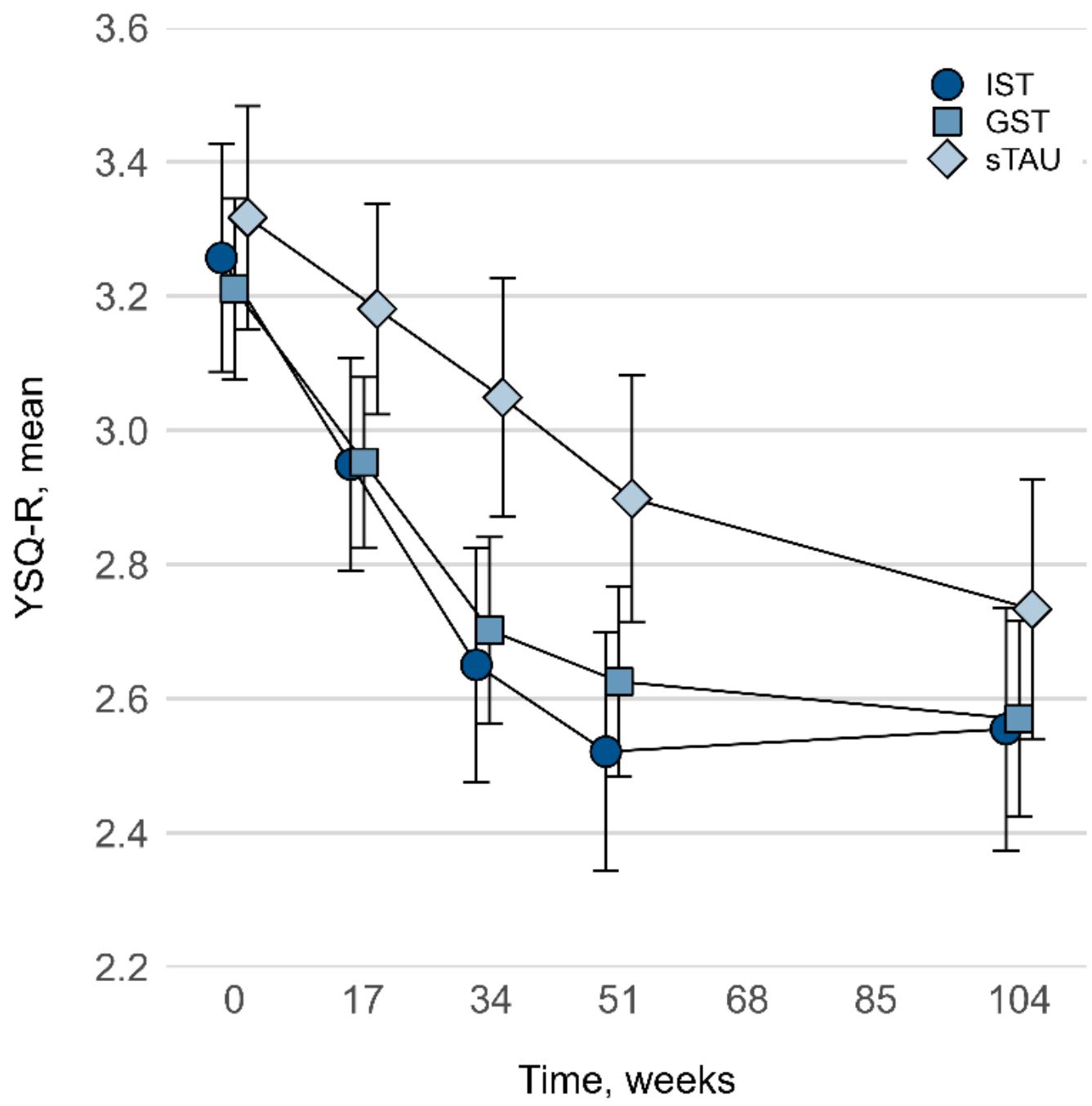

K. Schema modes – functional: SMI-2 functional

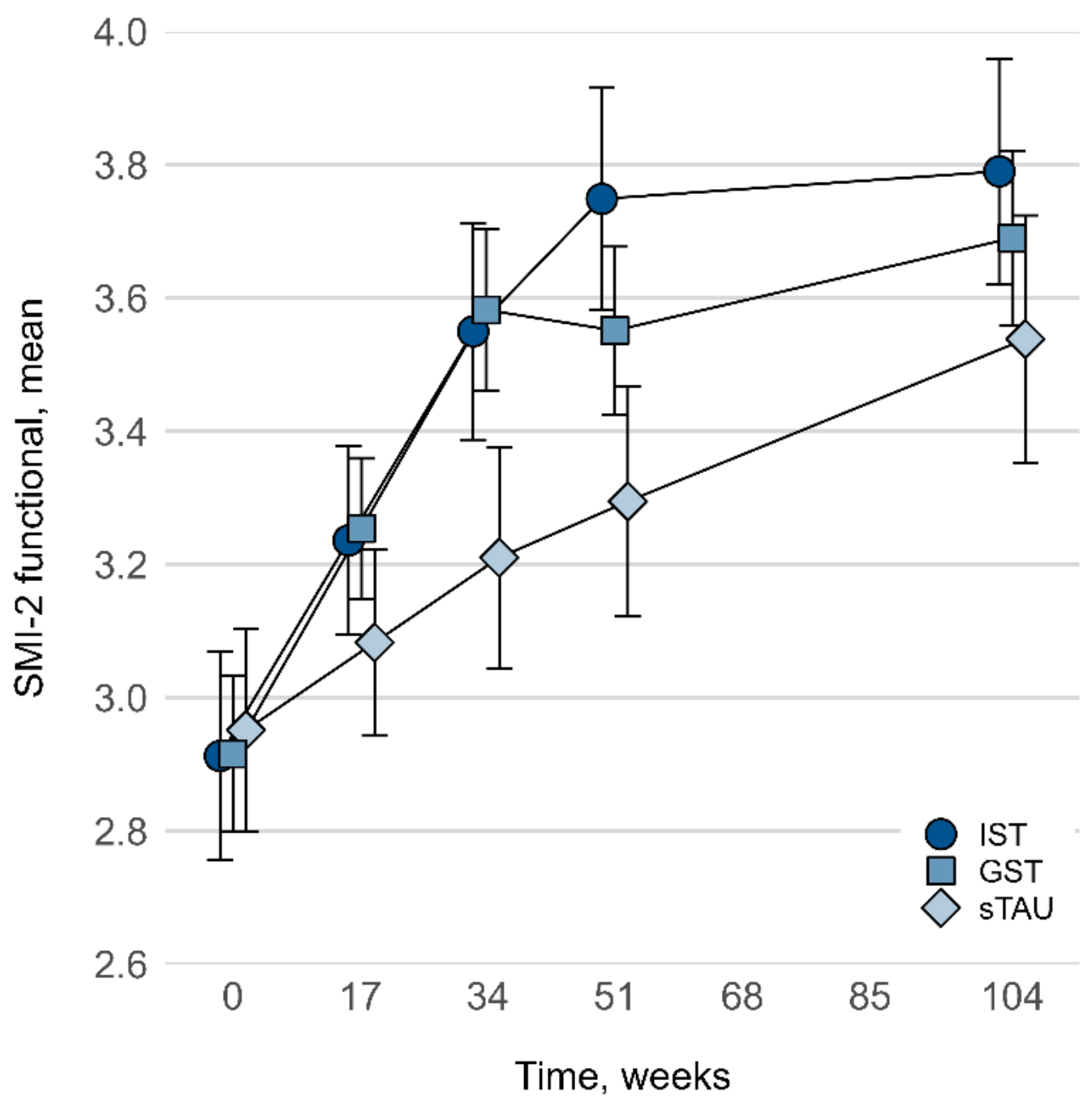

L. Schema modes – dysfunctional: SMI-2 dysfunctional

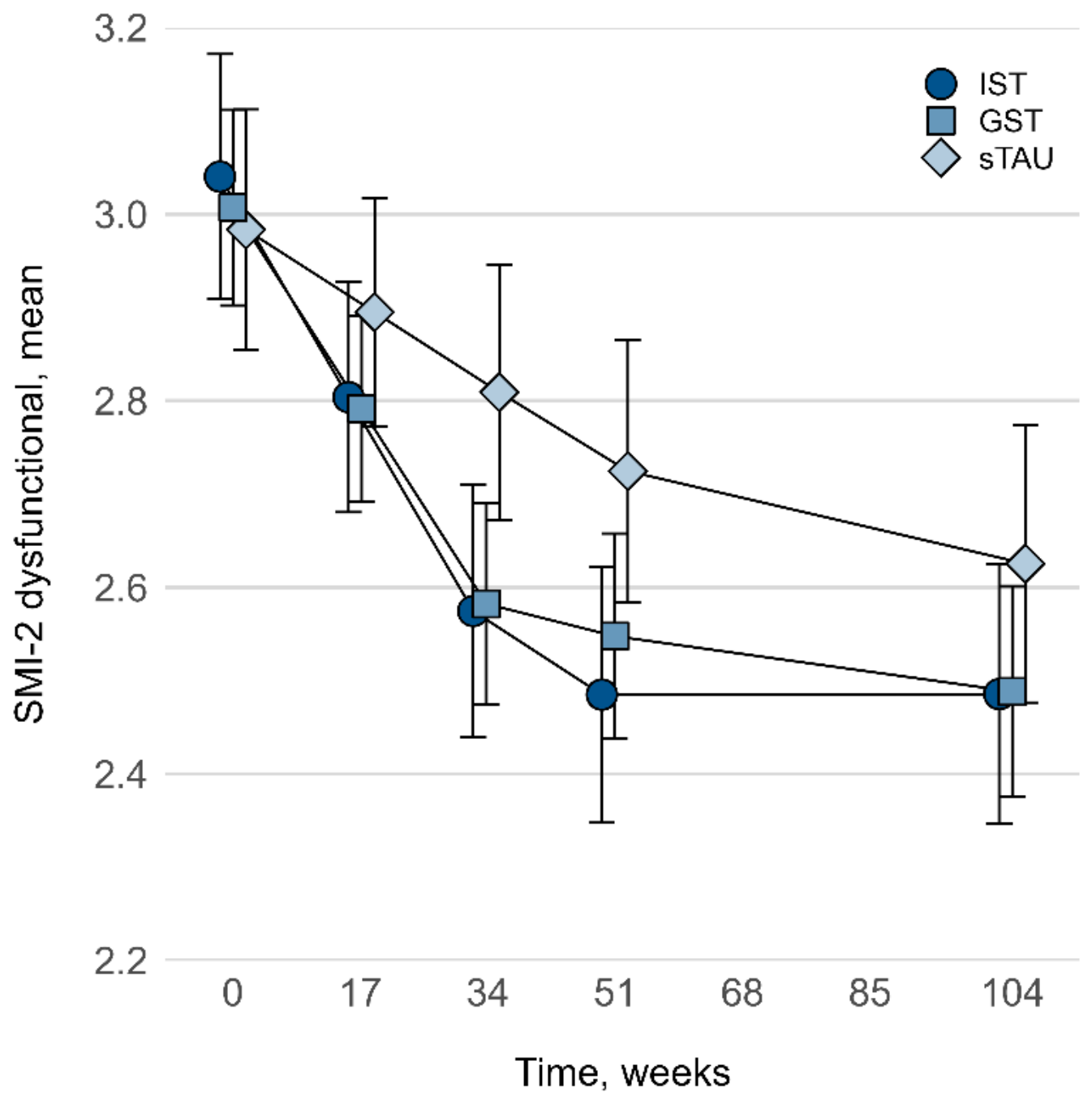

M. Sleep problems: SLEEP-50

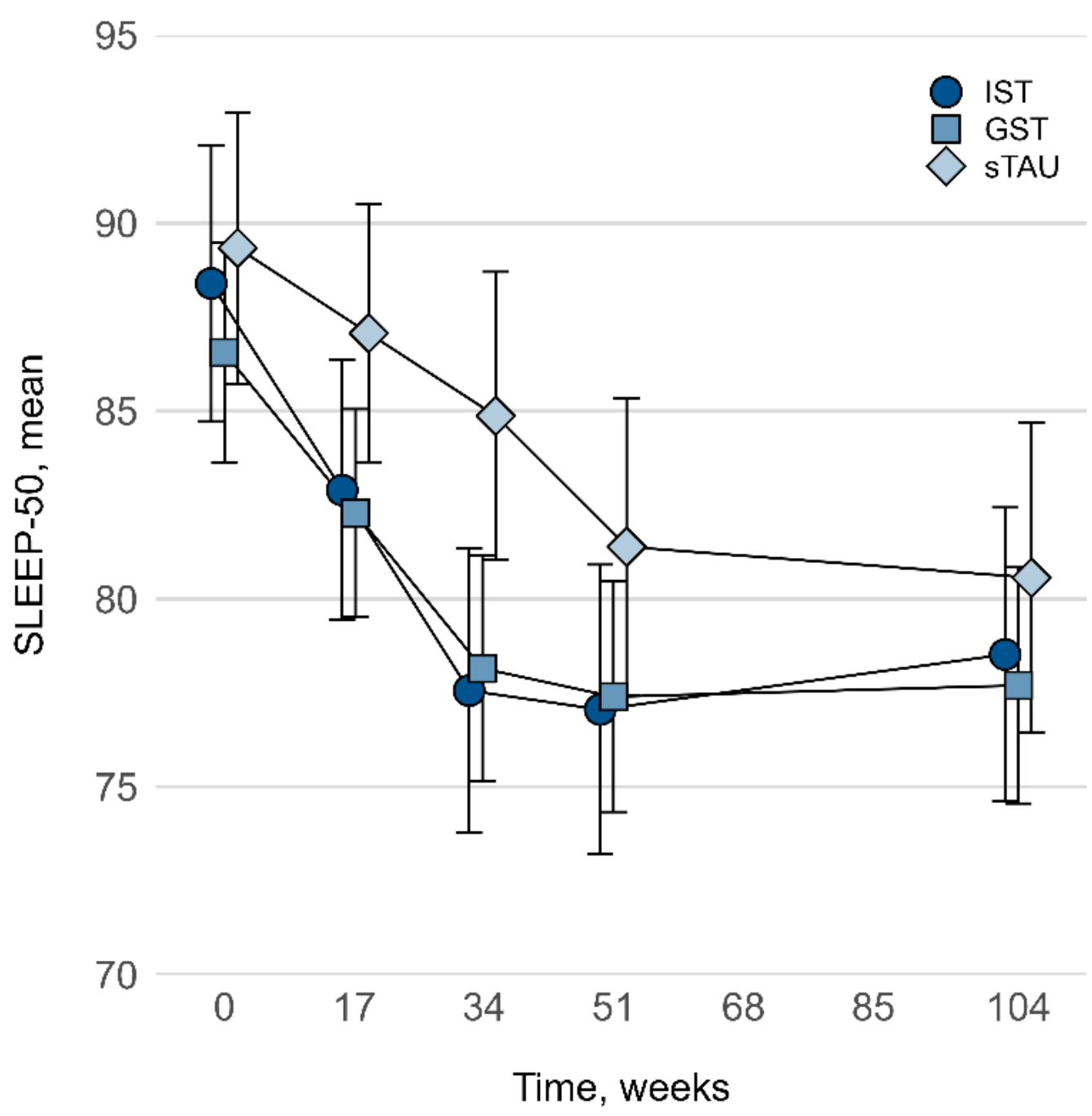

sReferences

1. Koo TK, Li MY. A Guideline of Selecting and Reporting Intraclass Correlation Coefficients for Reliability Research. *J Chiropr Med*. 2016;15(2):155-163. doi:10.1016/j.jcm.2016.02.012

2. Zarbock G, Farrell J, Schikowski A, et al. Group schema therapy rating scale - revised (GSTRS-R). Published online 2014.

3. Bastick E, Bot S, Verhagen SJW, et al. The Development and Psychometric Evaluation of the Group Schema Therapy Rating Scale – Revised. *Behavioural and Cognitive Psychotherapy*. 2018;46(5):601-618. doi:10.1017/S1352465817000741

4. Young J, Fosse G. *Schema Therapist Competency Scale for Individual Therapy Sessions*. 2008.

5. Hollon SD, Evans MD, Elkin I, Lowery A. System for rating therapies for depression. Published online 1984.

6. Bamelis LLM, Evers SMAA, Spinhoven P, Arntz A. Results of a multicenter randomized controlled trial of the clinical effectiveness of schema therapy for personality disorders. *J Psychiatry*. 2014;171(3):305-322.

7. Perepletchikova F, Treat TA, Kazdin AE. Treatment integrity in psychotherapy research: Analysis of the studies and examination of the associated factors. *J Consult Clin Psychol*. 2007;75(6):829-841. doi:10.1037/0022-006X.75.6.829

8. Baljé AE, Karch JD, Greeven A, et al. Avoidant Personality Disorder Severity Index: Dimensional structure and psychometric properties. *Pers Individ Dif*. 2023;213:112268. doi:10.1016/j.paid.2023.112268

9. Verheul H, Arntz A, van Empelen C, Debets B, de Vries E,. Psychometric evaluation of the Obsessive Compulsive Personality Disorder Severity Index. Published online 2020.

10. Tese S, Arntz A. The reliability and validity of the Dependent Personality Disorder Severity Index. Published online 2019.

11. World Health Organization. *World Health Organization Disability Assessment Schedule WHODAS II*. 2000.

12. Üstün T, Kostanjsek N, Chatterji S, Rehm J. *Measuring Health and Disability: Manual for WHO Disability Assessment Schedule WHODAS 2.0.* World Health Organizaition ; 2010.

13. Federici S, Bracalenti M, Meloni F, Luciano J V. World Health Organization disability assessment schedule 2.0: An international systematic review. . *Disabil Rehabil*. 2017;39(23):2347-2380.

14. Derogatis LR, Melisaratos N. The Brief Symptom Inventory: an introductory report. *Psychol Med*. 1983;13(3). doi:10.1017/S0033291700048017

15. De Beurs E, Zitman FG. De Brief Symptom Inventory (BSI)*. *Maandblad Geestelijke Volksgezondheid*. 2006;61(12):120-137. www.pits-online.nl

16. The EuroQol group. EuroQol-a new faciliEuroQol-a new facility for the measurement of health-related quality of lifety for the measurement of health-related quality of life. *Health Policy (New York)*. 1990;16(3):199-208.

17. van Krugten FCW, Busschbach JJ V., Versteegh MM, Hakkaart-van Roijen L, Brouwer WBF. The Mental Health Quality of Life Questionnaire (MHQoL): development and first psychometric

evaluation of a new measure to assess quality of life in people with mental health problems. *Quality of Life Research*. Published online 2021. doi:10.1007/s11136-021-02935-w

18. Versteegh MM, Vermeulen KM, Evers SMAA, de Wit GA, Prenger R, Stolk EA. Dutch Tariff for the Five-Level Version of EQ-5D. *Value in Health*. 2016;19(4):343-352. doi:10.1016/j.jval.2016.01.003

19. Feng YS, Kohlmann T, Janssen MF, Buchholz I. Psychometric properties of the EQ-5D-5L: a systematic review of the literature. *Quality of Life Research*. 2021;30(3):647-673. doi:10.1007/s11136-020-02688-y

20. van Krugten FCW, Feskens K, Busschbach JJ V., Hakkaart-van Roijen L, Brouwer WBF. Instruments to assess quality of life in people with mental health problems: a systematic review and dimension analysis of generic, domain- and disease-specific instruments. *Health Qual Life Outcomes*. 2021;19(1):249. doi:10.1186/s12955-021-01883-w

21. Abdel-Khalek AM. Measuring happiness with a single-item scale. *Social Behavior and Personality: an international journal*. 2006;34(2):139-150.

22. Dickhaut V, Arntz A. Combined group and individual schema therapy for borderline personality disorder: A pilot study. *J Behav Ther Exp Psychiatry*. 2014;45(2):242-251. doi:10.1016/j.jbtep.2013.11.004

23. Dreessen L, Arntz A. The Personality Disorder Beliefs Questionnaire (short version). Preprint posted online 1995.

24. Arntz A, Dreessen L, Schouten E, Weertman A. Beliefs in personality disorders: a test with the Personality Disorder Belief Questionnaire. *Behaviour Research and Therapy*. 2004;42(10):1215-1225. doi:10.1016/j.brat.2003.08.004

25. Rosenberg M. *Society and the Adolescent Self-Image*. 1965.

26. Franck E, De Raedt R, Barbez C, Rosseel Y. Psychometric properties of the Dutch Rosenberg self-esteem scale. *Psychol Belg*. 2008;48(1):25-35. doi:10.5334/pb-48-1-25

27. Miskimins RW, Braucht GN. *Description of the Self*. 1971.

28. Molendijk ML, Bamelis L, van Emmerik AAP, Arntz A, Haringsma R, Spinhoven P. Word use of outpatients with a personality disorder and concurrent or previous major depressive disorder. *Behaviour Research and Therapy*. 2010;48(1):44-51. doi:10.1016/j.brat.2009.09.007

29. Roelofs J, Papageorgiou C, Gerber RD, Huibers M, Peeters F, Arntz A. On the links between self-discrepancies, rumination, metacognitions, and symptoms of depression in undergraduates. *Behaviour Research and Therapy*. 2007;45(6):1295-1305. doi:10.1016/j.brat.2006.10.005

30. Weertman A, Arntz A. Effectiveness of treatment of childhood memories in cognitive therapy for personality disorders: A controlled study contrasting methods focusing on the present and methods focusing on childhood memories. *Behaviour Research and Therapy*. 2007;45(9):2133-2143. doi:10.1016/j.brat.2007.02.013

31. Arntz A, Venhuizen ASSM, Bachrach N, Groot I. *Introversion Inventory*. 2021.

32. Groot IZ, Kneip RL, Bachrach N, Venhuizen ASSM, Grasman RPPP, Arntz A. Psychometric Properties of the Introversion Inventory in Patients with a Cluster-C Personality Disorder and a Comparison Group . *in submission*.

33. Rijkeboer MM. Young Schema Questionnaire - Revised. Published online 2012.

34. Young JE, Brown G. Young schema questionnaire-short form; Version 3. Psychological Assessment. Published online 2005.

35. Bamelis LLM, Renner F, Heidkamp D, Arntz A. Extended Schema Mode Conceptualizations for Specific Personality Disorders: An Empirical Study. *J Pers Disord*. 2011;25(1):41-58. doi:10.1521/pedi.2011.25.1.41

36. Young JE, Arntz A, Atkinson T, et al. *The Schema Mode Inventory (SMI)*. 2007.

37. Spoormaker VI, Verbeek I, van den Bout J, Klip EC. Initial validation of the SLEEP-50 questionnaire. *Behavioral Sleep Medicine*. 2005;3(4):227-246. doi:10.1207/s15402010bsm0304_4

38. Stroup WW., Milliken GA., Claassen EA., Wolfinger RDean. *SAS for Mixed Models : Introduction and Basic Applications*. SAS; 2018.

39. Morris SB. Estimating Effect Sizes From Pretest-Posttest-Control Group Designs. *Organ Res Methods*. 2008;11(2):364-386. doi:10.1177/1094428106291059

40. Jacobson NS, Truax P. Clinical significance: A statistical approach to defining meaningful change in psychotherapy research. *J Consult Clin Psychol*. 1991;59(1):12-19. doi:10.1037/0022-006X.59.1.12

41. Wibbelink CJM, Venhuizen ASM, Grasman RPPP, et al. Group schema therapy for cluster-C personality disorders: A multicentre open pilot study. *Clin Psychol Psychother*. 2023;6(30):1279-1302. doi:10.1002/cpp.2903
